# Effects of MgSO_4_ Alone or Associated with 4-PBA on Behavior and White Matter Integrity in a Mouse Model of Cerebral Palsy: A Sex- and Time-Dependent Study

**DOI:** 10.3390/ijms232415947

**Published:** 2022-12-15

**Authors:** Lou Legouez, Bérénice Le Dieu-Lugon, Shérine Feillet, Gaëtan Riou, Melissa Yeddou, Thibault Plouchart, Nathalie Dourmap, Marie-Anne Le Ray, Stéphane Marret, Bruno J. Gonzalez, Carine Cleren

**Affiliations:** 1 INSERM U1245 “Cancer and Brain Genomics”—Team “Genetics and Pathophysiology of Neurodevelopmental Disorders”, IRIB, 76000 Rouen, France; 2 INSERM U1234 “Pan’Ther”, Flow Cytometry Core—IRIB, 76000 Rouen, France; 3 Department of Neonatal Paediatrics and Intensive Care-Neuropediatric, CHU, Rouen Hospital, 76000 Rouen, France

**Keywords:** hypoxic–ischemic lesion, magnesium sulfate, 4-phenylbutyrate, sex, behavior, white matter oligodendrocyte differentiation

## Abstract

Cerebral palsy (CP) is defined as permanent disorders of movement and posture. Prematurity and hypoxia–ischemia (HI) are risk factors of CP, and boys display a greater vulnerability to develop CP. Magnesium sulfate (MgSO_4_) is administered to mothers at risk of preterm delivery as a neuroprotective agent. However, its effectiveness is only partial at long term. To prolong MgSO_4_ effects, it was combined with 4-phenylbutyrate (4-PBA). A mouse model of neonatal HI, generating lesions similar to those reported in preterms, was realized. At short term, at the behavioral and cellular levels, and in both sexes, the MgSO_4_/4-PBA association did not alter the total prevention induced by MgSO_4_ alone. At long term, the association extended the MgSO_4_ preventive effects on HI-induced motor and cognitive deficits. This might be sustained by the promotion of oligodendrocyte precursor differentiation after HI at short term, which led to improvement of white matter integrity at long term. Interestingly, at long term, at a behavioral level, sex-dependent responses to HI were observed. This might partly be explained by early sex-dependent pathological processes that occur after HI. Indeed, at short term, apoptosis through mitochondrial pathways seemed to be activated in females but not in males, and only the MgSO_4_/4-PBA association seemed to counter this apoptotic process.

## 1. Introduction

Prematurity, defined as a birth before 37 gestational weeks (GW), can disrupt brain development and lead to short- and long-terms disabilities. Very preterm infants, born between 24 and 32 GW, are at high risk of neurodevelopmental disease such as cerebral palsy (CP). CP is defined as nonprogressive syndromes of posture and motor impairments, causing activity limitations and accompanied by neurodevelopmental disorders such as cognitive or sensory deficits [1]. Several risk factors can lead to CP as hypoxia-ischemia (HI) or infection/inflammation [2,3]. The prevalence of CP is inversely correlated to gestational age [4], and boys are more vulnerable to develop CP than girls [5]. The most common type of brain injury in preterm neonates is diffuse white matter injury (WMI), referred to as periventricular leukomalacia. These alterations are often associated with gray matter neuronal/axonal deficits and are named “encephalopathy of prematurity” [6]. A window of white matter vulnerability is described between 24–32 GW [3]. Indeed, during this period, most oligodendrocyte cells are oligodendrocyte precursor cells (OPCs) and preoligodendrocytes, which are highly vulnerable to high extracellular glutamate concentration, inflammation and energetic depletion due to their membrane composition. Indeed, these cells express NMDA receptors (R), cytokines R and ATP R. Moreover, these cells are sensible to oxidative stress because superoxide dismutase is not yet functional [7]. Diffuse WMI is characterized by specific OPCs degeneration in the early phase after an insult, followed by a compensatory proliferation phase of the surviving cells that fail to differentiate into mature myelinating OLs. These phenomena lead to OPCs accumulation and to the disruption of myelination [8]. 

Animal models can reproduce some aspects of human perinatal brain lesions and behavioral impairments at short and long terms. In rodents like in humans, the pattern of brain histological damage depends on age. HI performed in mice between postnatal day 1 to 5 (P1–P5) reproduces some aspects of brain lesions observed in preterm infants born between 24–32 GW [9,10,11]. Moreover, some experimental studies highlight a sex-dependent response to HI and reproduce high interindividual variability regarding behavioral and/or histological parameters [9,12,13,14], reported by clinical studies.

Nowadays, only magnesium sulfate (MgSO_4_) administration to women at imminent preterm delivery reduces CP prevalence in preterm infants. Indeed, several retrospective observational studies of MgSO_4_, firstly used as a tocolytic, have demonstrated a protective effect of antenatal exposure in the event of premature birth [15]. In 2009, a meta-analysis of five randomized controlled trials (6145 children) revealed a 32% reduction in the risk of developing CP, in 2-year-old children after an antenatal MgSO_4_ exposure, with no deleterious effects for the fetus nor the mother [16]. Moreover, two clinical trials have evaluated MgSO_4_ long-term outcomes. These studies indicate that antenatal exposure to MgSO_4_ is not statistically associated with better neurological, cognitive or behavioral outcomes in children aged between 6 and 11 years [17,18]. However, in the French PREMAG cohort, a tendency to decrease grade repetition is highlighted in the MgSO_4_ group [18]. Today, the FIGO (Fédération Internationale des Gynécologues et Obstétriciens, London, UK) working group recommends the usage of MgSO_4_ in women at risk of imminent birth under 30 weeks of gestation [19].

In the human body, magnesium ion is the second most abundant cation and is involved in more than 600 enzymatic reactions such as energy metabolism and protein synthesis [20]. Several in vitro and in vivo studies revealed that MgSO_4_ has anti-excitotoxic, anti-apoptotic and anti-inflammatory properties [21,22,23,24,25,26,27,28]. However, a few clinical and experimental studies highlight deleterious vascular effects of MgSO_4_ when administered at a high cumulative dose, never given in a neuroprotective trial in humans. Indeed, Mittendorf et al. (2006; [29]) reported that a high cumulative dose of MgSO_4,_ given for the treatment of pre-eclampsia in women, induces apoptosis and lenticulostriate vasculopathy in the fetus brain, as well as pediatric mortality. Moreover, a preclinical study showed that chronic MgSO_4_ treatment (600 mg/kg) in pregnant mice from gestational day 15 to gestational day 20 alters fetal cortical angiogenesis [30]. Indeed, during brain development, the vascular network supports oligodendrocytes and GABAergic interneurons migrations [31,32]. If altered, the vascular network may prevent cell integration in the cerebral parenchyma. 

Using the Rice–Vannucci model adapted to P5 mice [33], our previous research revealed, at short term, a complete prevention by MgSO_4_ of sensorimotor deficits induced by neonatal HI [13]. However, at long term, MgSO_4_ fails to prevent brain histological damages induced by neonatal HI in adolescent females, while it prevents these deficits in adolescent males. On the contrary, regarding behavior, MgSO_4_ fails to prevent behavioral deficits induced by neonatal HI in adolescent male mice but tends to prevent them in females [13]. Nonetheless, as previously explained, increasing the dose of MgSO_4_ is not a reasonable option because of potential deleterious effects. 

In order to prolong MgSO_4_ beneficial effects, a new project was developed consisting in associating MgSO_4_ to a second molecule with cell survival and anti-inflammatory and anti-oxidant properties, which could promote differentiation and myelination. The butyrate family was shown to present these properties after adult and neonatal HI in rodents [34,35,36,37]. Moreover, butyrates are short-chain fatty acids which easily cross the blood–brain barrier. Among butyrates, 4-Phenylbutyrate (4-PBA) was selected, since it is already administered to neonates to treat urea cycle disorders [38].

In the present study, using the Rice–Vannucci model on P5 pups, we address whether (i) 4-PBA alters MgSO_4_ neuroprotective effects at short term; (ii) the MgSO_4_/4-PBA association improves MgSO_4_ long-term effects; (iii) the MgSO_4_/4-PBA association promotes OL differentiation; and (iv) the MgSO_4_/4-PBA association presents deleterious proper effects. Moreover, sex-dependent effects were systematically investigated.

## 2. Results

First of all, the effects of three different doses of 4-PBA were investigated: 120, 600 and 1200 mg/kg to determine the one to be used within the association MgSO_4_/4-PBA for the rest of the study. For this purpose, weight intake from P5 to P10; temperature monitoring, cerebral lesion size and maternal behavior were studied. 

Concerning the weight intake, one-way ANOVA revealed a difference between groups (Supplementary Figure S1A; F = 3.219, *p* = 0.0176). Tukey’s post hoc test indicates that only the MgSO_4_/4-PBA association with a 4-PBA dose of 1200 mg/kg induced a less important weight intake in HI pups (vs. Sham-PBS–PBS or HI-PBS–PBS; *p* < 0.05, −50%). In contrast, MgSO_4_/4-PBA association with a 4-PBA dose of 120 and 600 mg/kg didn’t modify pups weight intake in comparison to sham-PBS–PBS or HI-PBS–PBS pups (*p* > 0.05). 

Pups body temperature was monitored at 0, 20 min, 40 min, 1 h, 2 h and 24 h after 4-PBA injection (120, 600, 1200 mg/kg). Two-way ANOVA did not reveal any interaction between the two factors Time and Treatment (Supplementary Figure S1B; F(25, 214) = 0.914, *p* = 0.54), but shows an effect of the treatment (F(5, 214) = 28.55, *p* < 0.001) and an effect of time (F(5, 214) = 18.85, *p* < 0.001). This graph reveals that the HI-Mg–4-PBA 120 (dark square) tend to have more difficulties in recovering their body temperature compared with the others.

TTC staining was performed to evaluate tissue viability in HI pups when injected with the MgSO_4_/4-PBA association (120, 600 or 1200 mg/kg; Supplementary Figure S1C). Tissue integrity is preserved in HI pups injected with 4-PBA at 120 and 600 mg/kg, contrary to those who received 1200 mg/kg 4-PBA. It is important to notice that HI and treatments do not disturb maternal behavior (results not shown).

Based on these observations, the dose of 600 mg/kg 4-PBA was selected to form the MgSO_4_/4-PBA association used in this present study.

### 2.1. In Pups, MgSO_4_ and the MgSO_4_/4-PBA Association Prevent HI-Induced Sensorimotor Deficits at Short Term

For sensorimotor tests, since results are similar in male and female pups, all results are shown with pooled sexes. The same animals are used for all behavioral tests at short term. 

The negative geotaxis test was performed at P6 and P7. Since the results at these two timings are very close, P6 data are shown in Supplementary Figure S2A.

**Figure 1:**
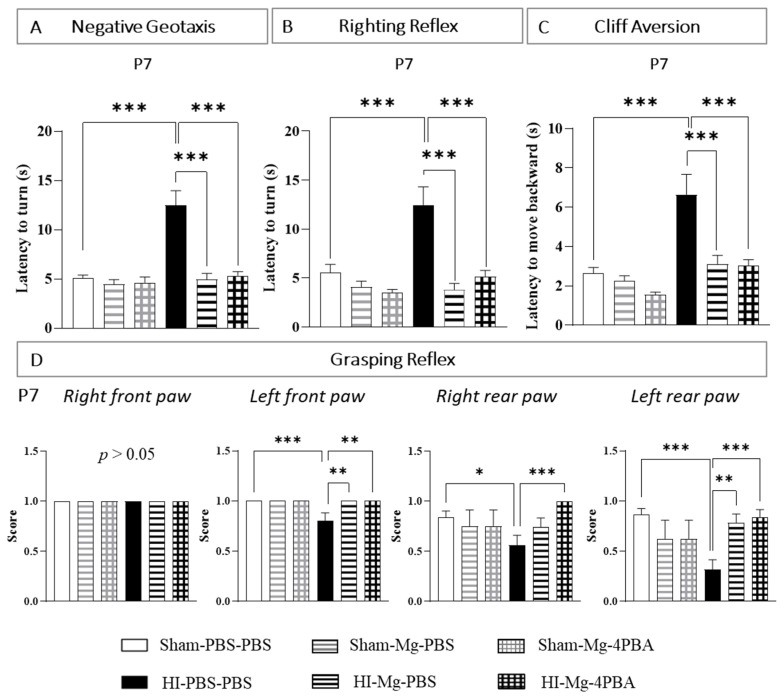
Effects of the neonatal HI, MgSO_4_ or of the MgSO_4_/4-PBA association on pups’ performances in the negative geotaxis (**A**), righting reflex (**B**), cliff aversion (**C**) and grasping reflex (**D**) tests at P7. Data are expressed as the latency (s) to turn (**A**,**B**) and to move backward (**C**), or as the presence or absence of the grasping reflex for each paw (score: 0 when reflex is absent, 1 when reflex is observed). (**D**) Sexes are pooled. Data are expressed as M ± S.E.M. *n* = 10–30 pups/group. Two-way ANOVA was performed followed by Tukey’s post-test. For grasping test, the Kruskal–Wallis test was performed followed by Dunn’s post-test. (* *p* < 0.05, ** *p* < 0.01, *** *p* < 0.001).

At P7, two-way ANOVA revealed an interaction between the two factors Surgery and Treatment (Figure 1A, F(2, 117) = 10.32, *p* < 0.001). According to Tukey’s post hoc test, neither MgSO_4_ nor the MgSO_4_/4-PBA association modified pups latency to turn at 180°, revealing the absence of proper effects (vs. Sham-PBS–PBS; *p* > 0.05). In contrast, neonatal HI alters pups’ performances (vs. Sham-PBS–PBS; *p* < 0.001, +150%). MgSO_4_ totally prevents HI-induced alterations (vs. HI-PBS–PBS, *p* < 0.001, −60%). Indeed, HI pups treated with MgSO_4_ display similar performances to Sham pups (vs. Sham-PBS–PBS; *p* > 0.05). Furthermore, the MgSO_4_/4-PBA association also totally prevents the HI-induced alterations (vs. HI-PBS–PBS: *p* < 0.001, −57%; vs. Sham-PBS–PBS: *p* > 0.05). MgSO_4_ and the MgSO_4_/4-PBA association prevent HI-induced alteration with the same efficacy (HI-Mg–PBS vs HI-Mg–4-PBA; *p* > 0.05). 

The righting reflex test was performed at P6 and P7. Since the results at these two timings are very close, P6 data are shown in Supplementary Figure S2B.

At P7, two-way ANOVA revealed an interaction between the two factors Surgery and Treatment (Figure 1B, F(2, 120) = 5.010, *p* < 0.0081). Tukey’s post hoc test shows that neither MgSO_4_ nor MgSO_4_/4-PBA association modifies pups’ latency to return on their four paws (vs. Sham-PBS–PBS; *p* > 0.05). HI pups display an increase in the latency to turn compared with Sham pups (vs. Sham-PBS–PBS, *p <* 0.001, +150%). MgSO_4_ totally prevents HI effects (vs. HI-PBS–PBS; *p* < 0.001, −69%). Indeed, HI pups treated with MgSO_4_ display similar performances to Sham pups (vs. Sham-PBS–PBS; *p* > 0.05). The MgSO_4_/4-PBA association also prevents HI-induced alterations (vs. HI-PBS–PBS, *p* < 0.001, −58%). Here, again, HI pups treated with the MgSO_4_/4-PBA association display similar performances to Sham pups (vs. Sham-PBS–PBS; *p* > 0.05). MgSO_4_ and the MgSO_4_/4-PBA association prevent HI-induced alteration with the same efficacy (HI-Mg–PBS vs. HI-Mg–4-PBA; *p* > 0.05)

The cliff aversion test was performed at P6, P7 and P10. Since the results at these three timings are very close, P6 and P10 data are shown in Supplementary Figure S2C.

At P7, two-way ANOVA revealed an interaction between the two factors Surgery and Treatment (Figure 1C, F(2, 126) = 3.681, *p* = 0.0279). Tukey’s Post hoc test shows that neither MgSO_4_ nor the MgSO_4_/4-PBA association change the time that pups use to be fully on the box (vs. Sham-PBS–PBS; *p* > 0.05). However, neonatal HI increases the latency to reach the platform (vs. Sham-PBS–PBS; *p* < 0.001, +166%). MgSO_4_ totally prevents these HI-induced deficits (vs. HI-PBS–PBS; *p <* 0.001, −53%; vs. Sham-PBS–PBS; *p* > 0.05). The MgSO_4_ /4-PBA association does not alter the prevention induced by the MgSO_4_ in HI pups (vs. HI-PBS–PBS; *p <* 0.001, −53%; vs. HI-Mg–PBS; *p* > 0.05, vs. Sham-PBS–PBS; *p* > 0.05).

The grasping reflex test was also performed at P6, P7 and P10. The presence or the absence of this reflex was searched for each paw. Since the results at these three timings were very close, P6 and P10 data are shown in Supplementary Figure S2D.

For the right front paw, the grasping reflex is present in every groups (Figure 1D).

At P7, regarding the left front paw, the Kruskal–Wallis test revealed a difference between groups (*p* = 0.0008). Dunn’s post hoc test revealed that HI alters this reflex (vs. Sham-PBS–PBS; *p* = 0.0004). MgSO_4_ totally prevents the HI (vs. HI-PBS–PBS; *p =* 0.0024; vs. Sham-PBS–PBS; *p* > 0.05). The MgSO_4_/4-PBA association also prevents HI-induced reflex alteration (vs. HI-PBS–PBS; *p* = 0.0018; vs. Sham-PBS–PBS, *p* > 0.05), with the same efficiency as MgSO_4_ alone (vs. HI-Mg–PBS; *p* > 0.05).

Concerning the right rear paw, the Kruskal–Wallis test revealed a difference between groups (*p* = 0.0085). According to Dunn’s post hoc test, neonatal HI significantly alters the grasping reflex (vs Sham-PBS–PBS; *p* = 0.0459). The score of the MgSO_4_/4-PBA association is not significantly different from the score of MgSO_4_ alone (vs. HI-Mg–PBS, *p* = 0.1956). However, only the MgSO_4_/4-PBA association significantly counters the HI-induced reflex alteration (vs. HI-PBS–PBS; *p* = 0.0009; vs. Sham-PBS–PBS, *p* > 0.05). 

Regarding the left rear paw, the Kruskal–Wallis test revealed a difference between groups (*p* = 0.0001). Dunn’s post hoc test revealed that HI induces a significant decrease in the grasping reflex (vs. Sham-PBS–PBS; *p* = 0.0001). MgSO_4_ prevents the HI effects (vs. HI-PBS–PBS; *p* = 0.0027), as well as the MgSO_4_/4-PBA association (HI-Mg–4-PBA vs. HI-PBS–PBS; *p* = 0.0003); this protection is total since no differences in grasping reflex are observed compared with Sham pups (HI-Mg–PBS vs. Sham-PBS–PBS; *p* > 0.05; HI-Mg–4-PBA vs. Sham-PBS–PBS; *p* > 0.05). MgSO_4_ and the MgSO_4_/4-PBA association prevent HI-induced alteration with the same efficacy (HI-Mg–PBS vs. HI-Mg–4-PBA; *p* > 0.05).

Overall, these data suggest that the MgSO_4_/4-PBA association does not alter the prevention induced by the MgSO_4_ at short term. Moreover, these data also indicate that the administration of MgSO_4_ and the MgSO_4_/4-PBA association does not induce deleterious proper effects on pups’ sensorimotor performances investigated, neither in males nor in females.

### 2.2. In Adolescent Mice, the MgSO_4_/4-PBA Association Prevents HI-Induced Motor Deficits

**Figure 2:**
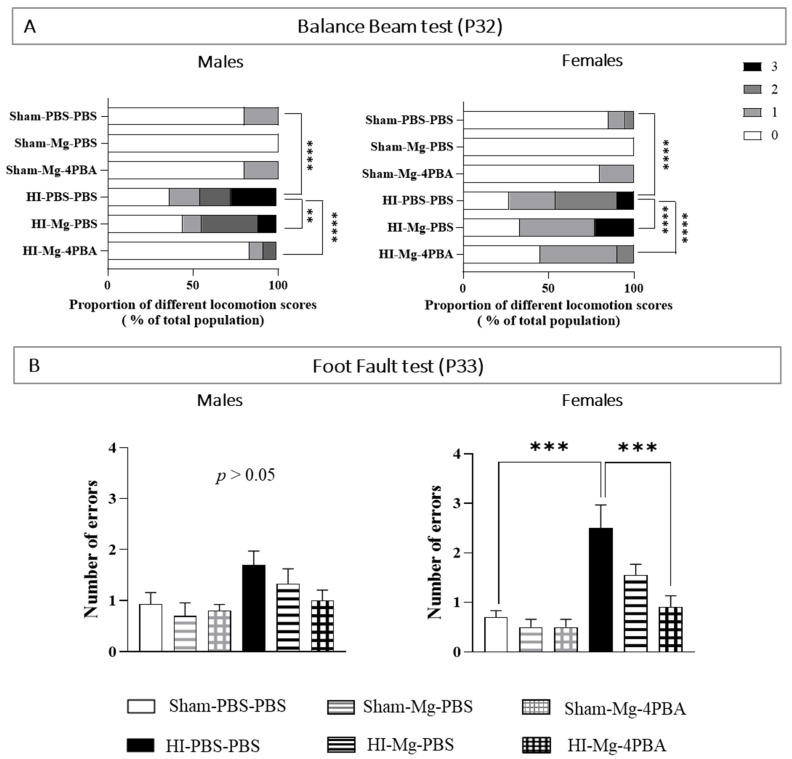
Effects of neonatal HI, MgSO_4_ or of the MgSO_4_/4-PBA association on motor abilities in adolescent male and female mice. (**A**)—Scores of locomotion in the balance beam test performed at P32. Data are expressed as proportion of scores. *n* = 5–14 males and 5–20 females/group. Chi2 test was executed. (**B**)—Number of errors on the foot-fault test performed at P33. Data are expressed as M ± S.E.M. *n* = 5–14 males and 5–20 females/group. Two-way ANOVA was performed followed by Tukey’s post-test (** *p* < 0.01, *** *p* < 0.001; **** *p* < 0.0001).

Motor abilities were tested in both adolescent males and females through the balance beam and the foot-fault tests performed, respectively, at P32 and P33 (Figure 2). Animals that realized these motor tests are the same as those that performed the behavioral tests at short therm. Since statistical differences were found between sexes in the balance beam test and in the foot fault test, sexes are presented separately. 

For the balance beam test (Figure 2A), Chi2 tests revealed a significant effect of HI and treatments in adolescent mice. Concerning the locomotion score of the balance beam test, in both male and female mice, neither MgSO_4_ nor the MgSO_4_/4-PBA association induced deleterious proper effects. HI increases high locomotion scores, reflecting an altered motor coordination compared with Sham mice (Males: Chi2 HI-PBS–PBS vs. Sham-PBS–PBS, df: 61.79, 3; ****, *p* < 0.0001; females: Chi2 HI-PBS–PBS vs. Sham-PBS–PBS, df: 62.33, 3, ****, *p* < 0.0001). MgSO_4_ in HI males partially prevents the HI-induced motor deficits (Chi2 HI-Mg–PBS vs. HI-PBS–PBS, df: 13.64, 3; **, *p =* 0.0034) by limiting the emergence of high locomotion scores. In HI females, MgSO_4_ partially prevents the HI-induced motor deficits (Chi2 HI-Mg–PBS vs. HI-PBS–PBS, df: 45.79, 3, ****, *p* < 0.0001,) by reducing light locomotion scores. In each sex, the MgSO_4_/4-PBA association totally prevents the HI effect (males: Chi2 HI-Mg–4-PBA vs. HI-PBS–PBS, 53.26, 3; ****, *p* < 0.0001; Chi2 HI-Mg–4-PBA vs. Sham-PBS–PBS, ns, *p* > 0.05; females: Chi2 HI-Mg-4PBS vs. HI-PBS–PBS, df: 33.70, 3, ****, *p* < 0.0001; Chi2 HI-Mg–4-PBA vs. Sham-PBS–PBS, ns, *p* > 0.05).

The foot-fault test was used to investigate a potential motor coordination deficit. In males, two-way ANOVA did not reveal any interaction between the two factors Surgery and Treatment (Figure 2B, F(2, 50) = 0.6276, *p* = 0.5380). However, in adolescent females, two-way ANOVA revealed interaction between the two factors Surgery and Treatment (F(2, 51) = 3.113, *p* = 0.05). According to Tukey’s post hoc test, no proper effects of MgSO_4_ or the MgSO_4_/4-PBA association are observed in female motor abilities. Neonatal HI induces a significant increase in the number of errors compared with Sham (vs. Sham-PBS–PBS; *p* < 0.001, +200%). In HI females, MgSO_4_ alone does not significantly prevent HI effects (vs. HI-PBS–PBS; *p* = 0.33), whereas MgSO_4_/4-PBA association totally prevents the HI-induced motor deficit (vs. HI-PBS–PBS; *p* < 0.001, −60%; vs. Sham-PBS–PBS; *p* > 0.05).

At long term, HI induces motor deficits that are sex-dependent and MgSO_4_ alone partially prevents the HI motor deficits, whereas it association with 4-PBA totally prevents it. Moreover, these data indicate that the administration of MgSO_4_ or MgSO_4_/4-PBA does not induce deleterious proper effects on motor abilities in adolescent mice.

### 2.3. In Adolescent Mice, MgSO_4_ or MgSO_4_/4-PBA Association Prevent HI-Induced Cognitive Behavioral Deficits

By using the social approach and social memory tests, the impact of neonatal HI and treatments on social mice behavior were evaluated. For social behavior evaluation, since the results are similar in males and females, all results are shown with sexes pooled. 

To study the social approach of a mouse, only one “stranger” mouse is put in one of the two cylinders. This mouse is called “Stranger 1” because it has never met the studied mice. A cylinder remains empty. 

In the Social Memory test, the studied mouse has two possibilities: having contact with the mouse already met the day before (the Stranger 1 of the social approach test becomes the familiar mouse in the social memory test), or having contacts with the “Stranger 2” mouse (that was never met before). 

**Figure 3:**
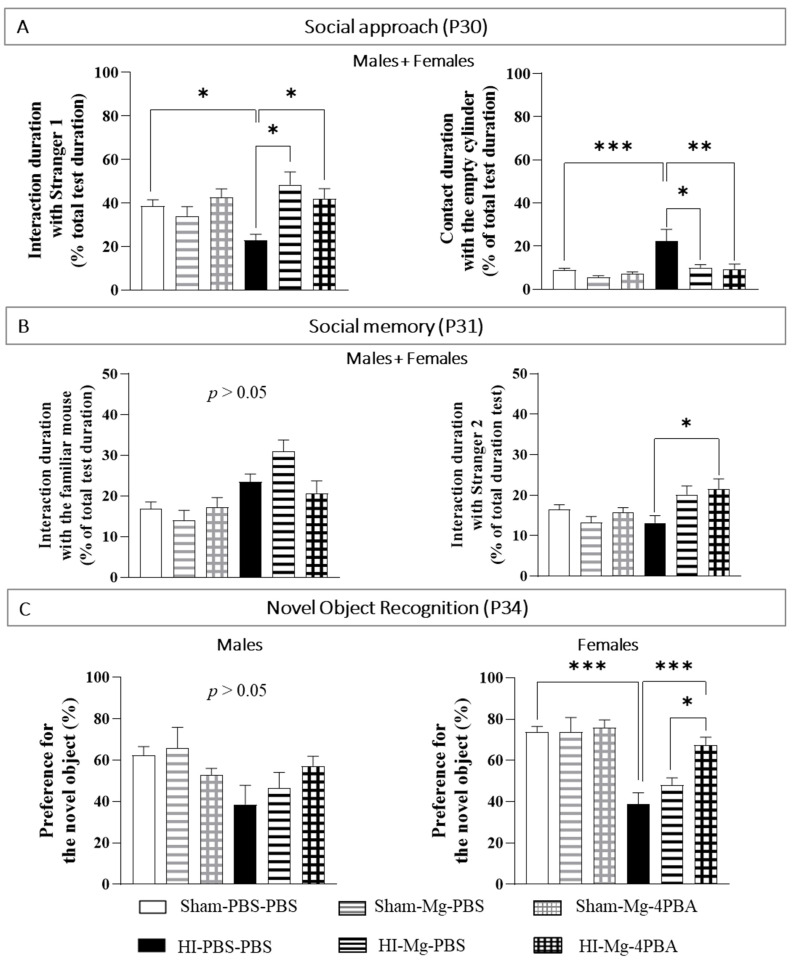
Effects of neonatal HI, MgSO_4_ or of the MgSO_4_/4-PBA association on cognitive capacities in adolescent male and female mice. (**A**)—Interaction duration with Stranger 1 and contact duration with the empty cylinder (% of total test duration) were measured with the social approach test at P30. *n* = 6–16 mice/group. Sexes are pooled. (**B**)—Interaction duration with the familiar mouse and interaction duration with Stranger 2 (% of total test duration) were measured with the social memory test, performed at P31. *n* = 6–16 mice/group. Sexes are grouped. (**C**)—Percentage of preference for the novel object at P34. *n* = 5–14 males and 5-20 females/group. Data are expressed as M ± S.E.M. Two-way ANOVA was performed followed by Tukey’s post-test (* *p* < 0.05, ** *p* < 0.01, *** *p* < 0.001).

Concerning the contact duration with the cylinder containing the Stranger 1 in the social approach test (Figure 3A), a two-way ANOVA revealed interaction between the two factors Surgery and Treatment (F(2, 85) = 5.860, *p* = 0.0041). Tukey’s post hoc test shows that MgSO_4_ or the MgSO_4_/4-PBA association do not induce deleterious proper effects on mice interaction abilities. HI mice spend less time interacting with the stranger than Sham mice do (vs. Sham-PBS–PBS; *p* = 0.0453, −42%). MgSO_4_ and the MgSO_4_/4-PBA association totally prevent HI-induced lack of interaction with the stranger (HI-Mg–PBS vs. HI-PBS–PBS, *p* = 0.014, +118%; HI-Mg–4-PBA vs. HI-PBS–PBS, *p* = 0.022, +90%; vs. Sham-PBS–PBS; *p* > 0.05). 

Concerning the contact duration with the empty cylinder in the social approach test, a two-way ANOVA revealed interaction between the two factors Surgery and Treatment (F(2, 82) = 4.559, *p* = 0.013). Tukey’s post hoc test shows that HI mice spend more time in contact with the empty cylinder (vs. Sham-PBS–PBS; *p* < 0.001, +175%). Moreover, MgSO_4_ and the MgSO_4_/4-PBA association significantly offset the HI-induced increase contact duration with the empty cylinder (HI-Mg–PBS vs. HI-PBS–PBS; *p* = 0.022, −54%, HI-Mg–4-PBA vs. HI-PBS–PBS; *p* = 0.0013, −59%). 

In the social memory test (Figure 3B), concerning the contact with the cylinder containing the familiar mouse, a two-way ANOVA did not reveal any interaction between the two factors Surgery and Treatment (Two-way ANOVA: F(2, 81) = 3.048, *p* = 0.06), but the factor Treatment induces an effect (F(1, 81) = 17.59, *p* < 0.001). On the other hand, concerning the contact with the cylinder containing the Stranger 2, a two-way ANOVA revealed an interaction between the two factors Surgery and Treatment (F(2, 67) = 5.545, *p* = 0.0064). Tukey’s post hoc test indicates that HI does not change the contact duration with the cylinder containing the Stranger 2 (vs. Sham-PBS–PBS; *p* > 0.05). Yet, the MgSO_4_/4-PBA association significantly increase contact duration with the cylinder containing the Stranger 2 in HI mice (vs. HI-PBS–PBS, *p =* 0.012; +65%).

The novel object recognition test was performed at P34 in order to assess memory abilities (Figure 3C). Since statistical differences were found between sexes in the novel object recognition, sexes are presented separately. 

In adolescent males, a two-way ANOVA does not reveal interaction between the two factors Surgery and Treatment (F(2, 49) = 2.139, *p* = 0.128). In contrast, in adolescent females, two-way ANOVA revealed an interaction between the two factors Surgery and Treatment (F(2, 58) = 4.364, *p* = 0.0172). Tukey’s post hoc test shows that neither MgSO_4_ nor the MgSO_4_/4-PBA association induce deleterious proper effects on female memory abilities. On the other hand, in adolescent females, neonatal HI induces long-term memory deficits (vs. Sham-PBS–PBS; *p* < 0.001, −49%). Only the MgSO_4_/4-PBA association totally prevents HI-induced memory deficits compared with HI females (vs. HI-PBS–PBS; *p* < 0.001, +76%, vs. Sham-PBS–PBS; *p* > 0.05). Moreover, HI females treated with the MgSO_4_/4-PBA association display an increase in the percentage of preference for the novel object compared with HI females treated with MgSO_4_ (vs. HI-Mg–PBS; *p* = 0.0216, +39%).

### 2.4. At Short Term, HI-Induced Cell Death Is Countered by Both MgSO_4_ and MgSO_4_/4-PBA Association

For molecular experiments, since no proper effects of the treatments were observed in any of the behavioral studies, only the HI groups were studied. 

**Figure 4:**
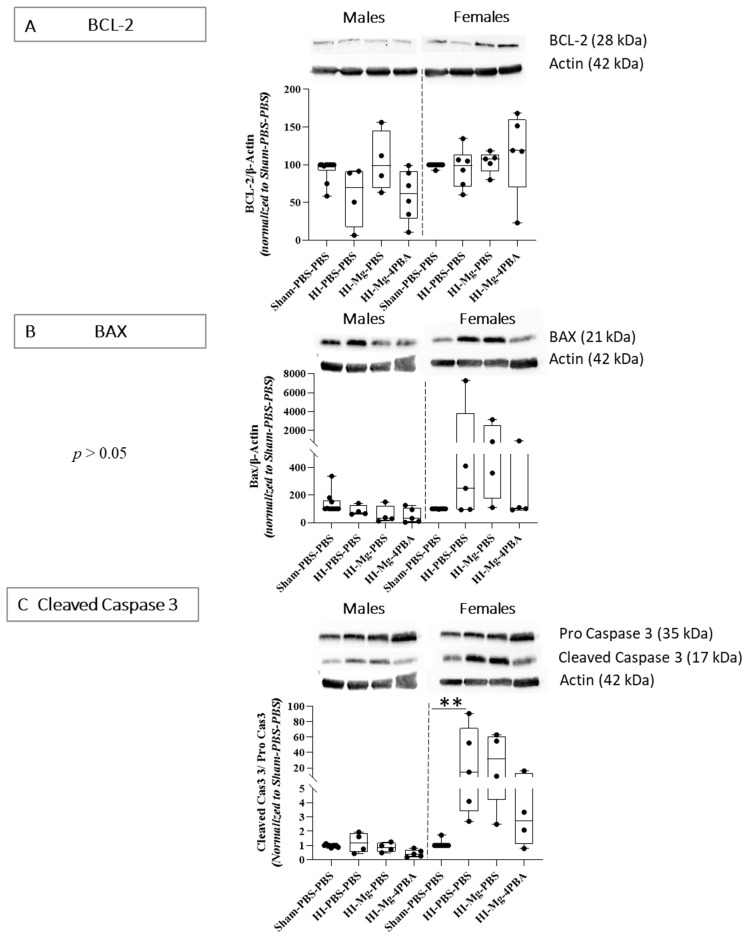
Effects of the neonatal HI, MgSO_4_ or of the MgSO_4_/4-PBA association on mitochondrial apoptotic proteins expression at P6. (**A**)—BCL-2 protein level 24 h after surgery. (**B**)—BAX protein level 24 h after surgery. (**C**)—Ratio of cleaved caspase 3 protein level over pro-caspase 3 protein level 24 h surgery. Data were normalized to the Sham-PBS–PBS group and expressed as median with min and max values, *n* = 4–10 males/group; 4–9 females/group. The Kruskal–Wallis test was performed followed by Dunn’s post hoc test (** *p* < 0.01).

Western blots from P6 brains, targeting apoptosis factors, particularly linked to the mitochondrial pathway, were performed (Figure 4). In males, concerning BCL-2 protein levels, the Kruskal–Wallis test revealed a difference between all groups (Figure 4A, *p* = 0.0274). However, Dunn’s post hoc test did not reveal any significant differences when groups were compared in pairs. The Kruskal–Wallis test did not reveal any differences between groups concerning the anti-apoptotic BCL-2 protein levels in females (*p* = 0.1461, Figure 4A). 

Concerning BAX protein levels, the Kruskal–Wallis test did not reveal any differences between groups in males and females (Figure 4B, *p* = 0.056 and *p* = 0.086, respectively). However, in females, there is a wide interindividual response in HI groups, with some individuals having a strong pro-apoptotic response. Visually, it seems that HI could increase the protein level of pro-apoptotic factor BAX. 

Concerning cleaved caspase 3 protein levels, the Kruskal–Wallis test did not reveal any differences between groups in males (Figure 4C, *p* = 0.06). However, in females, the Kruskal–Wallis test revealed a difference between groups (*p* = 0.027). Dunn’s post hoc test indicated that HI increases cleaved caspase 3 level (vs. Sham-PBS–PBS; *p* = 0.0046, +220%). At this time, the MgSO_4_/4-PBA association seems to reduce cleaved caspase 3 level in female HI mice, contrary to MgSO_4_ alone, but no significant result is observed. 

### 2.5. In P10 Pups, HI Induces a Massive Tissue Loss That Is Completely Prevented by MgSO_4_ and by the MgSO_4_/4-PBA Association

**Figure 5:**
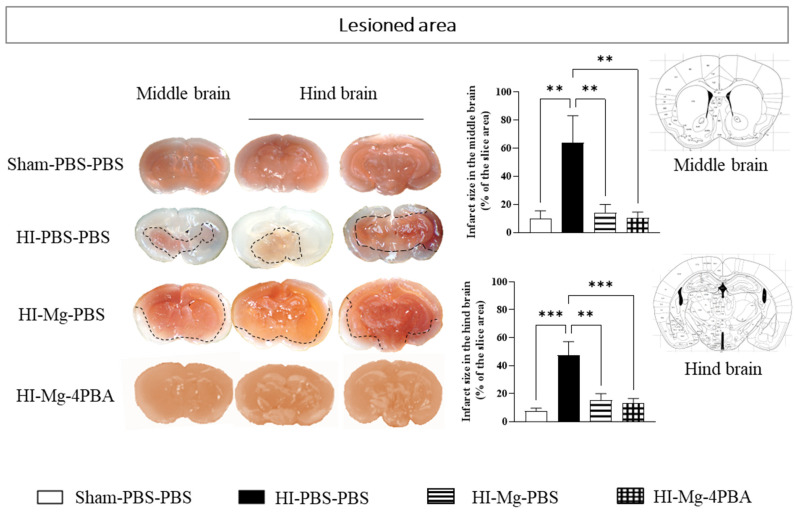
Effects of the neonatal HI with MgSO_4_ or MgSO_4_/4-PBA association on pups’ brain tissular viability at P10. Illustration of TTC staining of brain slices at the levels of the middle brain and the hind brain of P10 pups. Quantification of the lesioned area in HI pups treated with MgSO_4_ alone or with the MgSO_4_/4-PBA association. Sexes are pooled. Data are expressed as M ± S.E.M, *n* = 5–10 pups/group. One-way ANOVA was performed followed by Tukey’s post-test. (** *p* < 0.01, *** *p* < 0.001).

TTC staining was performed on brain slices from P10 pups (Figure 5) to evaluate the impact of neonatal HI on tissular integrity and the effects of treatments. Two regions were targeted: the middle brain with the large corpus callosum and the hind brain with the dorsal hippocampus. Concerning the infarct size in the middle and hind brain, one-way ANOVA revealed a difference between groups (F = 7.449, *p* = 0.0015 and F = 9.962, *p* = 0.0003, respectively). Tukey’s post hoc tests showed that neonatal HI induces a massive tissular loss in the middle (vs. Sham-PBS–PBS; *p* = 0.004, +742%,) and the hind (vs. Sham-PBS–PBS; *p* = 0.0003, +530%) brains. MgSO_4,_ as well as the MgSO_4_/4-PBA association, significantly prevent this HI-induced loss in the middle (HI-Mg–PBS vs HI-PBS–PBS; *p* = 0.008, −75%, HI-Mg–4-PBA vs HI-PBS–PBS; *p* = 0.0014, −83%) and the hind (HI-Mg–PBS vs HI-PBS–PBS; *p* = 0.0026, −66%, HI-Mg–4-PBA vs. HI-PBS–PBS; *p* = 0.0003, −72%) brains. Indeed, HI pups treated with MgSO_4_ or the MgSO_4_/4-PBA association displayed similar tissue viability to Sham (vs. Sham-PBS–PBS; *p* > 0.05).

### 2.6. Neonatal HI Changes the OPCs/Pre-OLs Repartition in the Corpus Callosum and in the Striatum 24 h after the Surgery, and Only the MgSO_4_/4-PBA Association Partially Prevents This Disorganization

**Figure 6:**
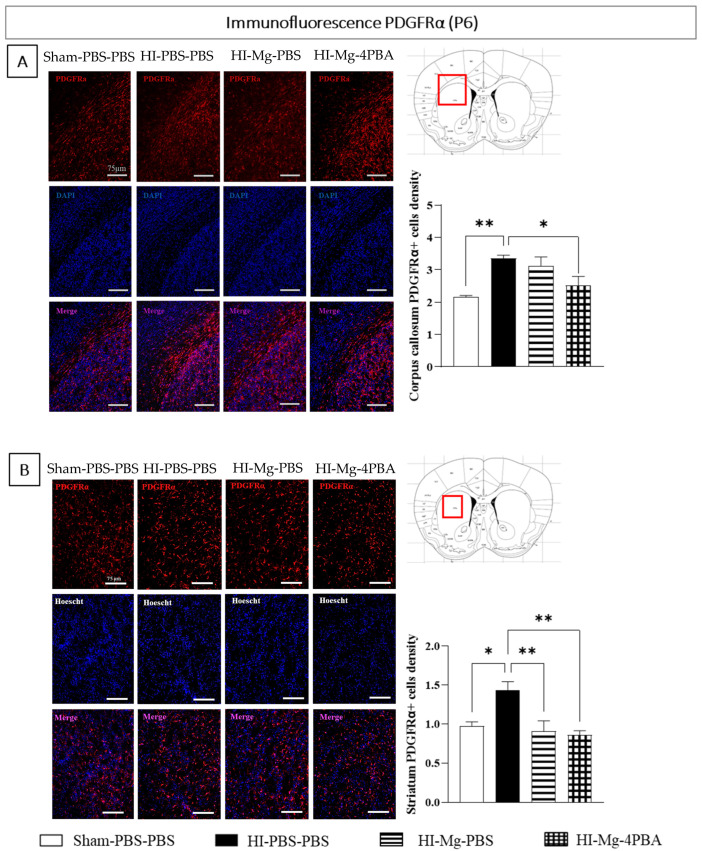
Effects of neonatal HI, MgSO_4_ or of the MgSO_4_/4-PBA association on PDGFRα density in the corpus callosum and striatum at P6. (**A**)—PDGFRα labeling immunofluorescence and quantification of PDGFRα+ cells density in the corpus callosum. (**B**)—PDGFRα labeling immunofluorescence and quantification of PDGFRα+ cells density in the striatum. Sexes are pooled. Data are expressed as M ± S.E.M, *n* = 5–6 pups/group. One-way ANOVA was performed followed by Tukey’s post-test. (* *p* < 0.05, ** *p* < 0.01).

For immunofluorescence experiments realized from P6 brains, since results are similar in male and female pups, all results are shown with pooled sexes (Figure 6). 

PDGFRα labeling was performed to evaluate the OPCs/Pre-OLs repartition in the corpus callosum, in the striatum of the ipsilateral hemisphere, 24 h after the surgery. 

Concerning the PDGFRα^+^ cell density in the corpus callosum, a one-way ANOVA revealed a difference between groups (Figure 6A, F = 7.949, *p* = 0.0016). Tukey’s post hoc test indicated that neonatal HI increases the PDGFRα^+^ cell density in the corpus callosum (vs. Sham-PBS–PBS; *p* = 0.0018, +44%). On this parameter, only the MgSO_4_/4-PBA association counters the HI effect (vs. HI-PBS–PBS; *p* = 0.0238, −20%). 

In striatum, a one-way ANOVA revealed a difference between groups (Figure 6B, F = 7.649, *p* = 0.0022) concerning the PDGFRα^+^ cell density. According of Tukey’s post hoc test, neonatal HI increases PDGFRα^+^ cell density in the striatum (vs. Sham-PBS–PBS; *p* = 0.0177, +48%). MgSO_4_ totally prevents these HI-induced deficits (vs. HI-PBS–PBS; *p* = 0.0065, −36%; vs. Sham-PBS–PBS; *p* > 0.05). The MgSO_4_/4-PBA association also totally prevents HI-induced alteration (vs. HI-PBS–PBS; *p* = 0.0030, −40%; vs. Sham-PBS–PBS; *p* > 0.05). 

Taken together, these data indicate that MgSO_4_ counters the OPCs/Pre-OLs accumulation induced by neonatal HI only in the striatum. However, the MgSO_4_/4-PBA association counters the HI-induced OL precursors in the striatum and in the corpus callosum. 

### 2.7. In Adolescent Mice, Neonatal HI Induces White Matter Alteration, and the MgSO_4_/4-PBA Association Partially Prevents This Alteration

**Figure 7:**
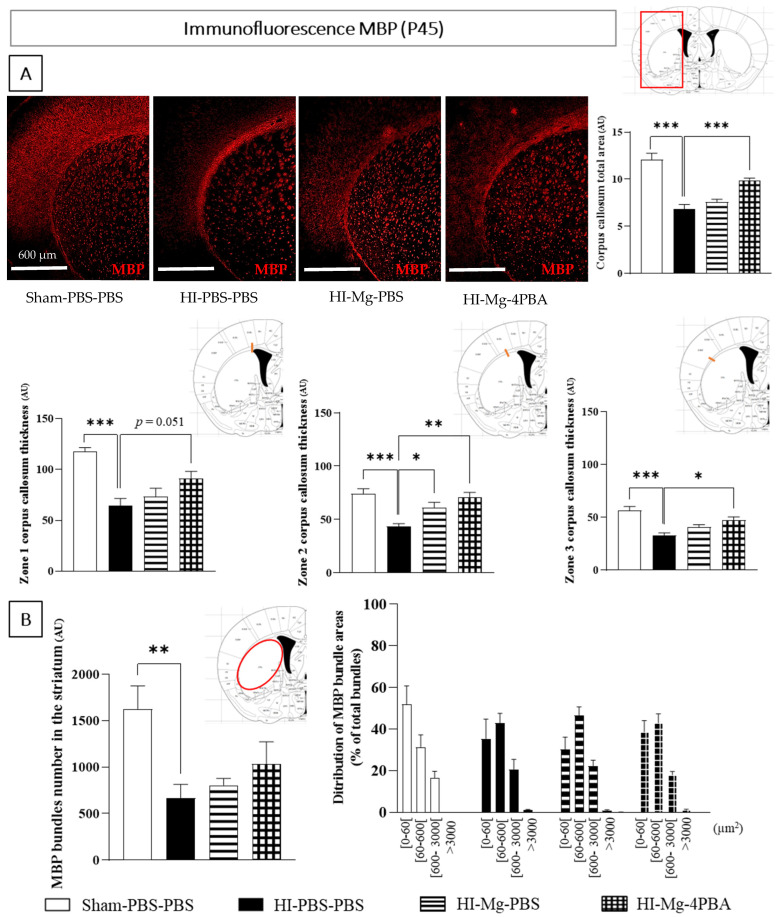
Effects of neonatal HI, MgSO_4_ or of the MgSO_4_/4-PBA association on myelin integrity (MBP) in the corpus callosum and the striatum of ipsilateral hemisphere, at P45. (**A**)—MBP labeling immunofluorescence and quantification of corpus callosum area and thickness in 3 zones containing axons that projects to the motor cortex (Zone 1), sensory cortex (Zone 2) and auditory cortex (Zone 3); AU: Arbitrary unit. (**B**)—Quantification MBP bundles number in the striatum and distribution of bundles sizes (µm^2^). Sexes are pooled. Data are expressed as M ± S.E.M, *n* = 5–6 pups/group. One-way ANOVA was performed followed by Tukey’s post-test (* *p* < 0.05, ** *p* < 0.01, *** *p* < 0.001). For bundle distribution, two-way ANOVA was performed; no significant result was obtained.

For immunofluorescence experiments realized from P45 mice brains, since results are similar in male and female pups, all results are shown with pooled sexes (Figure 7). 

Labeling a myelin sheath protein (MBP): The corpus callosum surface and thickness in the ipsilateral hemisphere were measured. For thickness, three zones were defined: the first one (zone 1) starts next to the ventricle and corresponds to axons that project into the motor cortex. The axon zone 2 projects into the somato-sensory cortex, and zone 3 contains axons that project into primary and associative auditory cortex.

Concerning the corpus callosum surface measurements, one-way ANOVA revealed a difference between groups (Figure 7A., F = 24.89, *p* < 0.001). Tukey’s post hoc test showed that neonatal HI decreases the corpus callosum total area (vs. Sham-PBS–PBS; *p* < 0.001, −48%). In HI mice, MgSO_4_ does not prevent HI effects (vs. HI-PBS–PBS; *p* > 0.05). Indeed, only the MgSO_4_/4-PBA association significantly counters the HI-induced decrease in corpus callosum total area (vs. HI-PBS–PBS; *p* = 0.0007, +33%).

For the corpus callosum thickness quantifications, one-way ANOVAs revealed differences between groups in the three studied zones (Zone 1: F = 12.71, *p* < 0.001; Zone 2: F = 10.68, *p* < 0.001; and Zone 3: F = 12.05, *p* < 0.001). According to Tukey’s post hoc tests, neonatal HI decreases the corpus callosum thickness in the three zones (vs. Sham-PBS–PBS; *p* < 0.001; zone 1: −45%, zone 2: −41%, zone 3: −41%) at long term. In HI mice, MgSO_4_ does not prevent the HI effects in zone 1 and 3 (vs. HI-PBS–PBS; *p* > 0.05), but it does significantly counter the HI-induced decrease in thickness in zone 2 (vs. HI-PBS–PBS; *p* = 0.0260, +27%). Concerning the corpus callosum thickness, the MgSO_4_/4-PBA association tends to prevent the HI-induced loss of thickness in zone 1 (vs. HI-PBS–PBS; *p* = 0.052; +40%) and significantly prevents the HI effect in zones 2 and 3 (vs. HI-PBS–PBS; *p* = 0.0025; *p* = 0.0141, respectively, Zone 2: +60%, Zone 3: +45%). 

With the same labeling (MBP), striatum fiber bundles were counted and sorted by size. One-way ANOVA revealed a difference in number of fiber bundles between groups (Figure 7B, F = 5.169, *p* = 0.0088). Tukey’s post hoc test showed that neonatal HI significantly decreases striatum fiber bundles number (vs. Sham-PBS–PBS; *p* = 0.0086, −58%). Neither MgSO_4_ nor the MgSO_4_/4-PBA association prevent the loss of white matter fibers in the striatum (vs. HI-PBS–PBS; *p* >0.05). 

Concerning the fiber bundles distribution by size, a two-way ANOVA did not reveal any interaction between the two factors Surface and Groups (F(12, 95) = 1.852, *p* = 0.0505). However, it revealed a strong effect of the factor Surface (F(4, 95) = 80.82, *p* < 0.001), revealing a different bundle distribution depending on the bundle area. 

Taken together, these data suggest that neonatal HI permanently disturbs white matter organization. Only the MgSO_4_ /4-PBA association partially prevents this disturbance. 

### 2.8. In Adolescent HI Mice, Oligodendrocyte Differentiation Is Facilitated by the MgSO_4_/4-PBA Association

**Figure 8:**
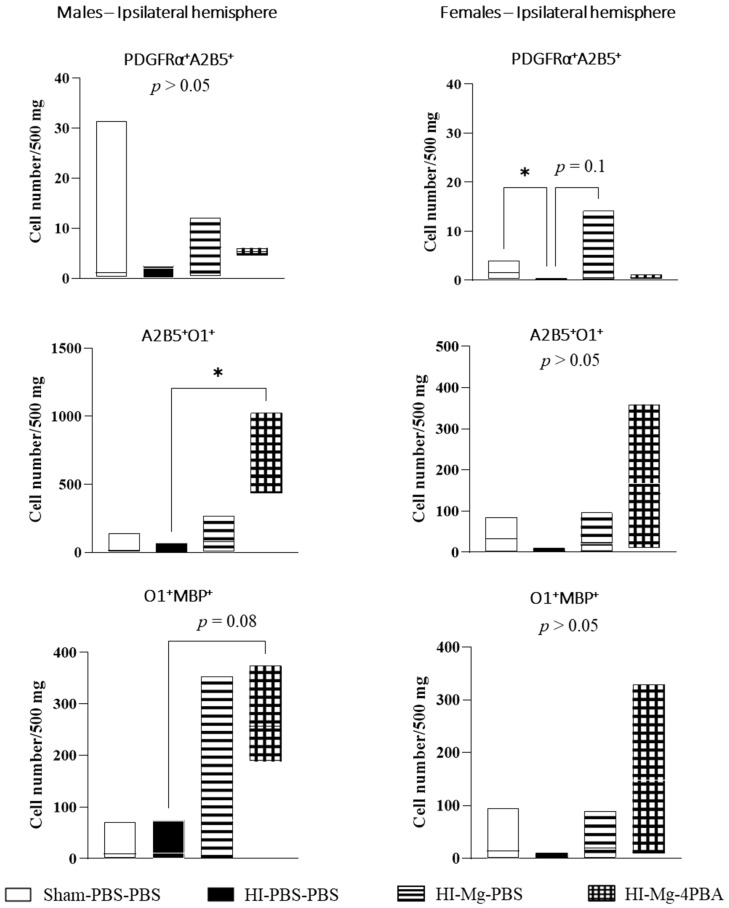
Effects of neonatal HI, MgSO_4_ or of the MgSO_4_/4-PBA association on oligodendroglial populations in the ipsilateral hemisphere in adolescent male and female mice at P45. Oligodendroglial populations were sorted by flow cytometry. Oligodendroglial populations were defined as PDGFRα+A2B5+ early oligodendrocyte progenitor cells, A2B5+O1+ premyelinating oligodendrocytes and O1+MBP+ mature myelinating oligodendrocytes. Data are expressed as median with min and max values, *n* = 3–5 males and 4–8 females/group. The Kruskal–Wallis test was performed followed by Dunn’s post-test (* *p* < 0.05).

In adolescent mice, the long-term effects of neonatal HI and treatments on OL differentiation were assessed. Using flow cytometry, early progenitor cells marked with PDGFRα^+^A2B5^+,^ premyelinating OL marked by A2B5^+^O1^+^ and mature myelinating OL marked by O1^+^MBP^+^ were counted (Figure 8).

In male adolescent mice, the Kruskal–Wallis test did not reveal any difference between groups concerning PDGFRα^+^A2B5^+^ cells (*p* > 0.05). However, the Kruskal–Wallis tests revealed differences in A2B5^+^O1^+^, O1^+^MBP^+^ cell numbers between groups (*p* = 0.0117 and *p* = 0.043, respectively). Dunn’s post hoc tests indicated that HI does not significantly change A2B5^+^O1^+^, O1^+^MBP^+^ cell number. However, the MgSO_4_/4-PBA association significantly increases A2B5^+^O1^+^ and tends to increase O1^+^MBP^+^ cell number (vs. HI-PBS–PBS; A2B5^+^O1^+^: *p* = 0.0236, +2300%, O1^+^MBP^+^: *p* = 0.08; +1024%).

In contrast, in females, concerning PDGFRα^+^A2B5^+^ cells, the Kruskal–Wallis test revealed a difference between groups (*p* = 0.0456). Dunn’s post hoc test shows that HI females display a decrease in PDGFRα^+^A2B5^+^ cell number compared with Sham females (vs. HI-PBS–PBS, *p* = 0.0165; −91%). Only MgSO_4_ in HI females tends to increase the PDGFRα^+^ A2B5^+^ cell number (vs. HI-PBS–PBS; *p* = 0.1, +175%). For the A2B5^+^O1^+^ and O1^+^MBP^+^ cell number, the Kruskal–Wallis test did not reveal any difference between groups (*p* = 0.0856 and *p* = 0.0848). 

Overall, this flow cytometry study shows that neonatal HI negatively impacts oligodendrocyte progenitors pool maintenance at long term but also oligodendrocyte differentiation and survival. MgSO_4_ tends to protect against progenitor loss in females but, when it is associated with 4-PBA, it tends to promote oligodendrocytes differentiation in both males and females. 

The main results of the study are summarized in Table 1.

## 3. Discussion 

Several clinical studies demonstrated that the administration of MgSO_4_ reduces CP prevalence in 2-year-old children born preterm [39]. However, underpowered studies reported only a trend to a decrease in child mortality in the 7-year follow-up of the ACTOMgSO_4_ trail [17], as well as a trend to a decrease in school difficulties in the 11-year follow-up of the PREMAG trial [18]. Moreover, while it is well-established that boys are more vulnerable to encephalopathy of prematurity [40], none of these clinical studies searched for a potential MgSO_4_ sex-dependent efficacy. Even so, since 2016, its use is recommended in several countries [41]. 

MgSO_4_ partial prevention at long term was also observed in a mouse model of perinatal brain lesion. Indeed, using the Rice–Vannucci model adapted to P5 mice, Daher et al. (2018; [13]) previously reported that, at short term, MgSO_4_ totally prevented HI-induced sensorimotor alterations in both male and female pups. However, at long term, MgSO_4_ partially prevented behavioral deficits and brain histological damage, such as white matter alterations, in a sex-dependent manner. Still, at long term, a mismatch was reported between histological and behavioral outcomes as observed in clinical settings. Therefore, in the present study, in an attempt to enhance and prolong MgSO_4_ neuroprotective effects, it was associated with 4-Phenyl butyrate (4-PBA). Indeed, Kim et al. (2007; [42]) reported beneficial effects from butyrates 24 h after a focal cerebral ischemia in adult rats, such as improvements in motor, sensory and reflex abilities. 

For this purpose, the longitudinal effects of both MgSO_4_ and the MgSO_4_/4-PBA association were compared through behavioral tests while investigating OL differentiation and searching for potential sex-dependent effects. 

### 3.1. No Deleterious Proper Effects of the Treatments at a Behavioral Level

The first relevant result of the present study is that, at short as well as at long terms, in non-HI mice, both the administration of MgSO_4_ alone or the MgSO_4_/4-PBA association did not induce any proper effects on mice behavioral abilities. Regarding MgSO_4_, this is in accordance with numerous clinical and experimental reports of its innocuity when administered in both types of studies at such doses. In fact, the dose of MgSO_4_ administered here (600 mg/kg, i.p.) leads to serum Mg^2+^ concentrations at a maximal level of 3.2 mmol/L at 30 min [23,43], in the range of levels reported to be neuroprotective (2.0–2.5 mmol/L at 1 h post MgSO_4_), and far below the toxic range (6 mmol/L in rats; [44]).

Regarding the MgSO_4_/4-PBA association, the absence of proper effects on short- and long-term behaviors concerning the parameters studied in the present study are promising. 

### 3.2. Short-Term Prevention of MgSO_4_ and MgSO_4_/4-PBA Association at a Behavioral Level

In infants with CP, difficulty with movement and coordination are the most common signs, along with delays in reaching motor developmental milestones [45]. Other frequent signs are, for instance, abnormal reflexes, asymmetric movement pattern, difficult walking and writing, vestibular imbalance and reduced fine motor skills. In addition, motor disorders may be associated with other neurodevelopmental disorders such as autism spectrum disorders (ASD). Indeed, ASD may be observed in up to 7% of children with CP who commonly present behavioral disorders [46]. Assessing cognition in children with motor impairments is not straightforward. Indeed, in children with severe speech and motor impairments, this is challenging; indeed, even small fine motor impairments might influence test scores negatively [47]. During adulthood, patients with CP present an increased risk of anxiety [48]. At a histological level, diffuse white matter injury, also called diffuse gliosis, is the most common form of injury in preterm children with CP [49]. Diffuse gliosis is characterized by pre-OL blockage of differentiation and accumulation that lead to disruption of myelination [8].

In this study, the Rice–Vannucci model in P5 pups mimics many of the behavioral and histological aspects of cerebral palsy that are listed above. Indeed, at short term, the sensorimotor abilities were evaluated by the negative geotaxis and the righting reflex tests, among others. In these two tests, MgSO_4_ pretreatment in HI mice totally prevented HI-induced sensorimotor deficits, as reported by Daher et al. (2018; [13]). It was also the case in another test, the cliff aversion test, which allows to evaluate labyrinth reflexes, strength and motor coordination when pups’ eyes are still closed. In HI pups, not only did the MgSO_4_/4-PBA association not alter the total prevention induced by MgSO_4_ in those three tests, but it also similarly induced a total protection. Moreover, in these three tests, the effects of MgSO_4_ and the MgSO_4_/4-PBA association in HI pups were equal in both sexes. 

In the present study, the deficits in front- or hindlimb use were evaluated at short term with the grasping reflex test, which determines the ability of pups in presenting the plantar/palmar grasping reflex [50]. On the whole, in both sexes and at the three times studied, the deficits induced by HI were more pronounced in the rear paws. This could be explained by the timing of myelination progression within the body, with nerves of the front paws being myelinated earlier than those of the rear paws. These deficits were totally prevented by both MgSO_4_ alone and by the MgSO_4_/4-PBA association, in both sexes.

The behavioral deficits described above at short term in mice may be imputable to cerebral lesions caused by the neonatal HI, which disturbed the neurodevelopment. To assess the impact of HI and treatments at short term on cerebral tissue viability, TTC staining was performed and revealed that neonatal HI induced a massive cell loss and impaired tissue integrity five days after neonatal HI. This loss of tissue viability was completely countered by MgSO_4_ alone, as reported earlier by [13]. Of great interest, the MgSO_4_/4-PBA association also totally prevented the HI-induced loss of cerebral tissue in a sex-independent manner. Such a result was suggested by Qi et al. (2004, [34]), who reported that 4-PBA reduces the infarct size after HI in adult mice. 

### 3.3. Diffuse White Matter Injury and Oligodendrocyte Precursor Accumulation

The pathological mechanisms involved in the emergence of brain lesions, mainly white matter lesions in the case of P5 neonatal HI, are rather well-established. Indeed, at this stage of brain development, oligodendrocyte precursors are particularly vulnerable to inflammation, glutamate concentration in extracellular space in the context of excitotoxicity and to energetic depletion [7,8,51]. This sensibility is granted by their membrane composition. Indeed, these cells present at their surface NMDA, cytokines and ATP receptors, which can initiate death programs such as necrosis, autophagy or apoptosis via the Ca^2+^ and Na^+^ entry into oligodendrocyte cells. This phase of OL precursor death is followed by a proliferative phase of the surviving cells that fail to differentiate into myelinating OLs. 

To investigate HI and treatments effects on white matter integrity at short term, PDGFRα labeling of P6 brain slices was performed. HI increased oligodendrocytes precursor density in the corpus callosum, as well as in the striatum. MgSO_4_ countered the HI-induced OL precursors density increase only in the striatum, while its association with 4-PBA allowed a total prevention in both the corpus callosum and the striatum. These results suggest that treatments at P5 of HI mice may prevent HI-induced dysregulated differentiation of expression factors implied in oligodendrocyte differentiation, such as at short term the proliferative of one PDGFRα. These may explain at least in part the better behavioral evaluations reported in the present study in mice that received the treatments. 

### 3.4. At Long Term, a Better Prevention of MgSO_4_/4-PBA Association at the Behavioral Level

These positive results are of major importance and drove the study of HI and treatments effects at a longer term. To evaluate HI and treatments effects on motor abilities in mice, the foot-fault test was performed at long term. Neonatal HI significantly increased the number of errors in females. MgSO_4_ failed to totally prevent these HI-induced deficits, while the MgSO_4_/4-PBA association completely prevented them. Then, in order to investigate HI and treatment effects on thin motor abilities and equilibrium, the balance beam test was performed. This test revealed that both males and females presented HI-induced deficits at long term, with imbalances compensated by particular types of walking, from small jumps to crab walk. MgSO_4_ alone partially reduced their occurrence in males and females, while the MgSO_4_/4-PBA association totally prevented them. Regarding cognitive abilities analyzed at long term, HI mice presented a significant impairment of memory abilities, which was not prevented by MgSO_4,_ whereas the MgSO_4_/4-PBA association significantly did. Huang et al. (2018, [36]) reported that sodium butyrate improves spatial learning and memory abilities in P30 rats that underwent neonatal white matter injury. 

To our knowledge, no study investigated social behavior in mice which underwent neonatal HI. The present study revealed that HI induced long-term social approach deficits. Moreover, these results highlight the fact that both MgSO_4_ and the MgSO_4_/4-PBA association totally prevented HI-induced deficits in social approach, with the same efficiency. Since the present study reports interesting results from cognitive tests performed at long term, it would be interesting to investigate HI and treatments effects through the Plus maze test, since reducing anxiety could favor brain plasticity after brain lesion by promoting compensatory mechanisms. 

### 3.5. Sex Dependent Effects of HI and Treatments

Sex differences are observable among children with CP. Indeed, it has been shown that preterm boys are more vulnerable to develop CP than preterm girls [40]. During childhood, Chounti et al. (2013, [52]) did not identify any statistical difference concerning motor deficit severity between boys and girls with CP. However, when CP subtypes are individually studied, boys are predominant in spastic and dyskinetic CP, usually with a hypoxic–ischemic insult in the neonatal history, whereas girls dominate among children with ataxic CP caused by cerebellar alteration. This observation would be in accordance with the notion that female periventricular neurons may be more resistant to energy depletion [53]. Moreover, boys are predominant in higher scores of manual motricity deficits. In the present study, HI also induced some sex-dependent effects, regarding motor and cognitive abilities at long term. Indeed, HI induced significant deficits in females regarding voluntary motricity, while it induced more severe scores in males in the balance beam test, which requires more equilibrium. Regarding cognitive deficits, HI induced memory deficits only in females in the present study. 

These different observations between males and females concerning some motor and cognitive abilities may be underlined by sex-dependent pathophysiological responses to HI. These sex-dependent processes could already occur at short term during lesion genesis. To evaluate this hypothesis, the mitochondrial apoptotic pathway was studied via Western blot labeling of the pro-apoptotic protein, BAX and the anti-apoptotic protein, BCL-2. When BAX is dimerized at the mitochondrial membrane, it forms a canal that permits pro-apoptotic proteins to get out toward the cytoplasm. This leads to apoptosome formation and to caspase 3 activation through its cleavage. The present study revealed that 24 h after HI, there was a significant increase in caspase 3 activation in females, while in males there was no activation at all. Moreover, in females, there was a strong interindividual variability. In male HI mice, no changes in BAX protein levels were observed. Altogether, these data reaffirm that HI-induced apoptotic pathway activation is sex dependent [54]. Indeed, an in vitro study demonstrated that from E17, in rat XY and XX neurons exposed to a cytotoxic agent, apoptosis mainly proceeds via a pathway dependent on apoptosis-inducing factors in XY neurons versus a mitochondrial pathway and caspases in XX neurons [55]. More recently, Charriaut-Marlangue et al. (2017, [56]) reported that there are sexual dimorphism pathways after neonatal hypoxia–ischemia. Indeed, regarding apoptosis, it is mainly induced by PARP activation in males, while the caspase 3 activation dominates in females.

Regarding treatment effects on HI-induced apoptosis, the association of MgSO_4_/4-PBA tended to prevent pro-apoptotic HI effects. Qi et al. (2004; [34]) have shown that 4-PBA countered cell death after adult HI in mice. 4-PBA is a chemical chaperon, getting rid of misconfigured proteins. Probably by this function, 4-PBA was shown to decrease ER stress and cell death in oligodendrocytes after adult hypoxic optic neuropathy [37], as well as in oligodendrocyte cell lines expressing mutated proteolipid protein (PLP), which is a major myelin protein [57]. Indeed, when mutated or misconfigured, this protein leads to the degeneration of oligodendrocyte and to the development of myelin disorders.

In order to investigate whether behavioral deficits could be associated with white matter alterations, HI and treatments effects on OLs different subpopulations were investigated by flow cytometry in mice when adults. In females, HI induced a decrease in OPC cell number and tended to decrease pre-Ols and mature myelinating Ols. On the contrary, MgSO_4_ tended to increase OPCs number, while the MgSO_4_/4-PBA association tended to increase the number of both pre-myelinating OLs and mature myelinating OLs. In males, HI did not change the number of each oligodendroglial sub-population but MgSO_4_/4-PBA increased premyelinating OLs and tended to increase mature myelinating OLs. Since these experiments were realized using the total ipsilateral hemisphere, it would be interesting to directly focus on brain structures strongly impacted by HI, such as the striatum or the corpus callosum through brain tissular microdissection. 

### 3.6. Long-Term Effects of HI and Treatments on White Matter Integrity

To visualize if these modifications of oligodendroglial sub-population proportions after HI and treatments were associated with modifications in white matter integrity, MBP labeling was performed in the present study. Neonatal HI induced myelination deficits, observable through the decreased corpus callosum surface and thickness and through the decrease in the striatum white matter fiber bundles. However, Silva et al. (2019; [58]), in CYFIP1-deleted oligodendrocyte cells in culture, have demonstrated that MBP has to be localized in oligodendrocyte processes to induce regular myelination. Thus, studying MBP location in OLs coupled with electronic microscopy would be of great interest. In the present study, concerning treatments effects on HI-induced alterations in the corpus callosum and striatum, MgSO_4_ did not prevent the HI-induced white matter alterations. On the contrary, the MgSO_4_/4-PBA association partially prevented myelination disruption in the corpus callosum. Huang et al. (2018; [36]) have shown that sodium butyrate promotes oligodendrocyte differentiation after a neonatal HI in P2 rat, preventing HI-induced myelination disruption at long term. 

In physiological condition, oligodendrocytes differentiation and survival are controlled by many epigenetic mechanisms, among them histone methylation and acetylation [59,60,61], which lead to the inhibition or promotion of positive/negative differentiation factors, depending on OLs differentiation stage. Huang et al. (2018, [36]) have showed that seven days after a neonatal HI in P2 rat pups, the acetylation of histone H3 within oligodendrocytes was decreased, and differentiation and maturation of OLs were inhibited. This was countered by sodium butyrate, which is a histone deacetylase inhibitor (iHDAC) from the butyrate family, as 4-PBA, which also inhibits class I and IIa histone deacetylases [62]. Therefore, to deeper understand the impact of neonatal HI, MgSO_4_ and of the MgSO_4_/4-PBA association on oligodendrocyte differentiation, modulations of chromatin compaction and epigenetics marks of OPCs and pre-OLs should be investigated.

### 3.7. Limitations

One limitation of the present study is that the experimental model by itself is long-lasting, reducing the feasibility of investigating the direct effects of molecules. Another limitation concerns the timings when the molecular effects of HI and treatments were studied. Indeed, it would have been interesting to evaluate these effects between the short- and long-term experiments that were performed. This was not achievable because of the necessity to keep mice alive to perform this longitudinal study until long term. 

### 3.8. Conclusions

Overall, this study shows that at short term, the MgSO_4_/4-PBA association does not alter the total prevention induced by MgSO_4_ alone on (i) HI-induced behavioral deficits, which was already reported, (ii) nor for more discrete behavioral deficits analyzed in the present study. This total prevention can partly be explained by the promotion of OL precursors differentiation after HI and by anti-apoptotic properties of the MgSO_4_/4-PBA association. At long term, the MgSO_4_/4-PBA association enhances and/or prolongs MgSO_4_ prevention at behavioral and cellular levels, especially by countering some white matter alterations. Taken together, these results are very encouraging and indicate the possibility to associate several molecules in order to develop more efficient neuroprotective strategies in a sex-dependent manner. Thus, by associating MgSO_4_ with 4-PBA, several neuroprotective mechanisms with different targets may result in additive effects.

## 4. Materials and Methods

### 4.1. Animals and Housing

National Marine Research Institute (NMRI) mice, purchased from Janvier Lab (France) were housed at 22 °C with food and water ad libitum and with a 12 h light/dark cycle (lights on from 7 a.m. to 7 p.m.). Male and female mice are handled every day from P5 to P45. Males and females are weaned and put in separate cages at P21. Animal handling is performed according to the recommendations of the European Communities Council directives (2010/63/UE) and the French national legislation, with an authorization delivered by the French Ministry of Education and Research (agreement no. 01680.02/2014, October 13, 2014; APAFIS#221362019092013438607 v4 from Ministère de l’Agriculture et de la Pêche). At P6, P10 and P45, mice are euthanized respecting ethical methods (P6 and P10: decapitation after profound anesthesia with isoflurane 5%; P45: cervical elongation under profound anesthesia with isoflurane 5%, VETFLURANE^®^). The number of animals is minimized as much as possible. Animal welfare is insured throughout the study.

### 4.2. Mouse Model of Hypoxic–Ischemic Insult

**Figure 9:**
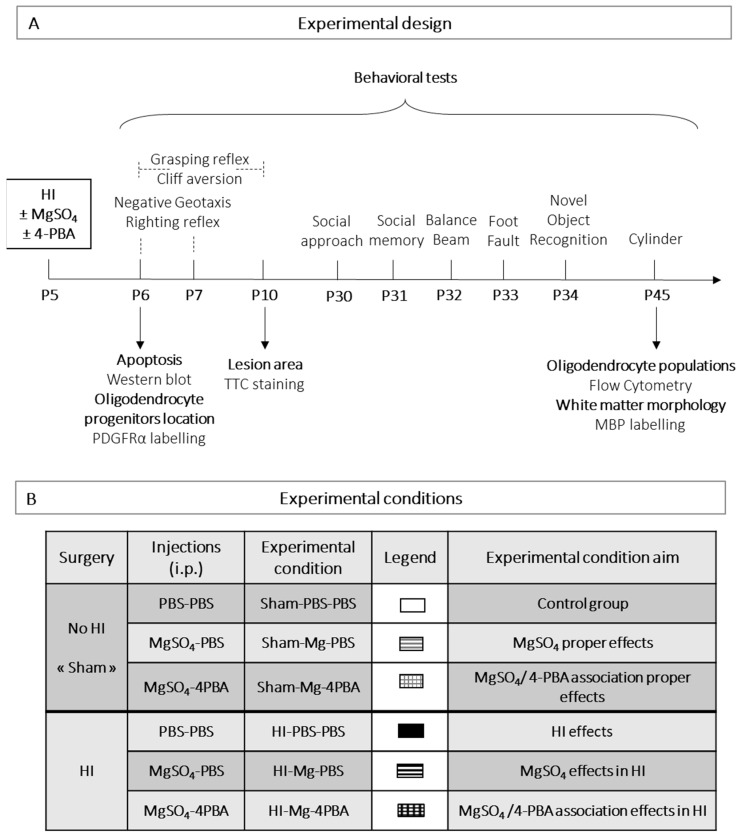
Experimental design of the study (**A**) and experimental conditions (**B**). HI: Hypoxia-Ischemia; P5: Postnatal day 5; i.p.: intraperitoneally; PBS: Phosphate buffer salin; MgSO_4_: Magnesium sulphate; 4-PBA: 4-Phenylbutyrate; Association: 4-PBA + MgSO_4_.

The mouse model of hypoxia–ischemia is based on the Rice–Vannucci model [33], adapted to P5 mice as previously described [13]. Concisely, the left carotid artery is permanently ligated in half of P5 anesthetized pups (isoflurane 4% for the induction and 2% for the maintenance, VETFLURANE^®^). One hour after ligation, MgSO_4_ (600 mg/kg; [28]) or phosphate buffered saline (PBS) is injected intraperitoneally (i.p.). One hour after injections, pups are placed in hypoxic chamber at 36 °C with O_2_ 8% for 45 min. Sham animals (surgery without ligation) are separated from their dams and placed in normoxic condition. One hour after hypoxic event, 4-PBA (600 mg/kg) or PBS is injected i.p. The design of the study and experimental groups are described in Figure 9.

### 4.3. Behavioral Evaluation

To evaluate behavioral performances at short and long terms, the same animal cohort is used, and the same sequence of behavioral tests is followed for each animal.

#### 4.3.1. Mother and Pups Well-Being Checking after Surgery and Treatments

Mother behavior towards pups is observed during and after the Rice–Vannucci procedure every day until weaning. Mothers’ stress levels are checked by searching repetitive grooming behaviors, compulsive cage cleaning and disinterest for pups. 

Pups feeding and pups weight intake are closely observed, especially during 4-PBA dose investigation. For that, pups are weighed each day, from surgery (P5) to P10. For each pup, P5 weight is subtracted from P10 weight to obtain the weight intake (Δ weight = P10 weight-P5 weight). Then, for each condition, weight intakes are averaged. Moreover, pups’ body temperatures are monitored before 4-PBA injection and 20 min, 40 min, 2 h and 24 h after the 4-PBA injection with a thermal probe (Harvard apparatus). 

#### 4.3.2. Behavioral Evaluations in Pups

In pups, behavioral tests are conducted from P6 to P10 to evaluate motor and sensory abilities. As previously described by Daher et al. (2017; [23]), the righting reflex test is performed to evaluate pups’ sensorimotor ability and the negative geotaxis test to evaluate vestibular and motor coordination at P6 and P7. In the righting reflex test, the time needed for a pup placed on its back on a flat surface to turn over on its 4 limbs is recorded. The cut-off time per trial is set at 60 s. Each value is the mean of the 3 successive trials separated by a 10 s delay. After the test, pups are placed back in their home cage. In the negative geotaxis test, the time needed for a pup placed head down on a 30° inclined surface to turn 180° is recorded. Here, again, the cut-off time per trial is set at 60 s. Each value corresponds to the mean of 2 trials, separated by a 10 s delay.

The grasping reflex test is performed at P6, P7 and P10 in order to assess front- or hindlimb troubles. As described by Feather-Schussler and Ferguson (2016; [50]), each paw is tested by using a no-sharp and thin object. The presence or the absence of the grasping is recorded with the score 1 when the pup has the grasping reflex and 0 when it does not.

The cliff aversion test is performed at P6, P7 and P10 in order to evaluate labyrinth reflexes, motor strength and coordination. As described by Feather-Schussler and Ferguson (2016; [50]), the pup is placed on a 17 cm-high box with its snout and front limbs overlooking the void. The time needed to fully move back on the top of the box is recorded.

#### 4.3.3. Behavioral Evaluations in Adolescent Mice

In adolescent mice, from P30 to P45, motor and cognitive tests are performed. In chronological order, social approach and memory social test are performed at P30 and P31, the balance beam test is performed at P32, the foot-fault test at P33, the novel object recognition test at P34 and the cylinder test at P45. 

#### 4.3.4. Social Approach and Memory 

The social approach test is performed at P30. The tested mouse is placed in a rectangular device (90 × 43 cm) containing two cylinders (h = 13 cm, d = 8 cm) placed equidistantly from the edges (22 cm from edges). The cylinder permits to overcome aggressive behaviors. Only the tested mice’s social approach behavior is measured. Initially, the cylinders are empty, and the animal freely explores the device for 5 min. Then, during the 5 min test phase, a stranger mouse “Stranger 1” is added in the left cylinder. Both times spent in contact with “Stranger 1” and in contact with the empty cylinder are measured. 

Twenty-four hours after the social approach test, at P31, the tested mouse is replaced in the rectangular device for 5 min. “Stranger 1” of the social approach test becomes the familiar mouse in the social memory test. In the right cylinder, which is empty during the social approach test, an unfamiliar mouse (“Stranger 2”) is placed. Interaction durations with the “familiar” mouse or with the “Stranger 2” mouse are measured. 

#### 4.3.5. Balance Beam Test 

The balance beam test is performed at P32 in order to highlight fine motor coordination alterations [63]. A fine wooden beam (66 cm long and 0.6 cm wide) is used. Mice are placed at one end of the beam and the home cage at the other end. Together, the number of imbalances and the type of walking give a score, as described in Supplementary Table S1.

#### 4.3.6. Foot-Fault Test 

The foot-fault test is performed at P33 in order to evaluate the motor coordination deficits, as previously described by Daher et al. (2018; [13]). The horizontal ladder consists of two Plexiglas walls in which irregularly spaced metal bars are inserted. The home cage is placed at one end of the ladder. A habituation phase is performed 24 h before the test. Two trials are performed for each mouse. The mouse places their paws on the bars while moving along the apparatus. An error is recorded when a paw falls or slips between two bars. Each value corresponds to the mean of number of errors committed during the two trials.

#### 4.3.7. Novel Object Recognition Test 

The novel object recognition is performed at P34 in order to evaluate memory deficits, as previously described by Daher et al. (2018; [13]). Animals are placed in a cage (43 × 27 × 20 cm) similar to the home cage. In the first session, two identical conical objects (6 × 1 cm) are used, whereas in the second session, one of the two objects is replaced by a rectangular object (2 × 1 × 6 cm). During the first session, mice freely explore the two identical objects for 10 min. Twenty-four hours later, mice with one familiar object are added the novel object for 5 min. The time spent exploring each object is recorded when mice sniff and/or interact with the objects from a distance ≤1 cm. In order to measure the mouse memory performance, a preference index is calculated as follows: 

Preference index = ((time exploring the novel object—time exploring the familiar object)/total exploration time) × 100.

A zero value indicates no preference for either object. Values from 1% to 100% indicate various degrees of preference for the novel object, whereas negative values indicate a preference for the familiar object. 

### 4.4. Cylinder Test

The cylinder test highlights the rodent’s spontaneous forelimb use to evaluate the sensory-motor function in injury models that cause forelimb use asymmetry [64,65]. In this test, the mouse is placed in a glass cylinder, and the number of times it gets up and lands and touches the cylinder wall and base are measured. The wall/base touches are subsequently counted for left, right, or both paws in slow-motion-recorded videos (Panasonic Camera, HC-V250). The results are expressed as the percentage of each paw use relative to the total number of touches. 

### 4.5. Histological Evaluation by TTC Staining

TTC, a metabolic assay to determine tissue viability, is performed on brain slices at P10. Brains are sectioned into 1 mm-thick slices in a coronal plane (Zivic Labs). Slices are stained with TTC 2% (Sigma, 93140-50G) in the dark at room temperature for 30 min, followed by paraformaldehyde (PFA) 4% at 4 °C for 2 h. Slices are observed using a camera (Infinity 2, Lumenera Corparation, Ottawa, Canada). ImageJ Fiji software (National Institute of Health, Bethesda, Maryland, MD, USA) is used for quantitative analysis of lesion areas at 3 levels of anteriority from the most rostral section, according to Paxinos et al. (2007; [66]): −2.79 mm as level 1, −4.11 mm as level 2 and −4.71 mm as level 3. TTC is used to identify metabolically active and inactive tissues. TTC is reduced to 1,3,5-triphenylformazan (TPF), a red dye, in living tissues by a number of dehydrogenases, enzymes involved in many metabolic pathways, while it remains white in necrotic tissues because these same enzymes are degraded or denatured. 

### 4.6. Evaluation of White Matter Integrity at Short and Long Term by Immunostaining 

Brains are extracted and immersed in 4% paraformaldehyde (PFA, Sc-281692) for 1 h for the P6 brains and 2.5 h for P45 brains. They are then transferred in a cryoprotective solution (sucrose/PBS 30%). When brains sink in the solution, they are frozen in −30 °C isopentane and stored at −80 °C until completion of 20 µm sections for P6 brains and 40 µm sections for P45 brains. 

The sections are incubated for 45 min in a permeabilizing buffer (PBS, BSA 1%, triton X-100 0.3%) at room temperature, then incubated overnight at 4 °C with the primary antibody (1/200 in permeabilizing buffer) directed against PDGFRα (R&D system AF1062, Goat polyclonal) for P6 sections and MBP (Abcam Ab7349, Rat monoclonal) for P45 slices. After 7 rinses of 3 min each in PBS, the sections are incubated with secondary antibodies (1/400 in permeabilizing buffer; For PDGFRα labeling: Alexa Fluor 594 Donkey anti-goat, Life technology A11058; For MBP labeling: Alexa Fluor 594 Donkey anti-Rat, Invitrogen) directed against the primary antibodies and bound to a fluorochrome for 1.5 h in the dark with slow shaking. The slices are rinsed and incubated with a fluorescent DNA intercalating agent to label nuclei (Hoechst, 1/5000 in PBS, Sigma Aldrich). Slices are observed by using the Leica DMI 6000B microscope and the images are obtained using the Metamorph^®^ Leica AF software. 

Specificity and auto-fluorescence controls were carried out by replacing the primary antibodies with permeabilization buffer alone or by not adding primary and secondary antibodies. Monitoring of the stability of the fluorochrome over time (illuminated during 6 h) was also carried out during the development phase. 

The density of PDGFRα positive (PDGFRα+) cells in the corpus callosum (Bregma: 3.27 mm, [66]) and in the striatum is measured using ImageJ Fiji software. For each animal, the density of PDGFRα^+^ cells is averaged over two successive sections. 

The area of the corpus callosum (Bregma: 0.02 mm, [67]) and its thickness in 3 different zones, at P45, are measured using ImageJ software. The counting and sorting of white matter fiber bundles in the striatum are also realized by using ImageJ software.

### 4.7. Evaluation of Apoptosis Factors Protein Levels by Western Blot

Total proteins are extracted from P6 brains and dosed by colorimetry (Eppendorf, biophotometer). One-hundred micrograms of protein are run on 12% SDS-polyacrylamide gels and transferred to nitrocellulose membranes (midi-size nitrocellulose; Trans-Blot Turbo^TM^, Bio-Rad^®^). The membranes are subsequently blocked using 5% milk (diluted in Tween-Tris saline buffer 20%) and probed with Rabbit anti-BCL 2 (1/1000 in milk 5%, Abcam Ab2568, Rabbit polyclonal), Rabbit anti-BAX (1/1000 in milk 5%, Abcam Ab32503, Rabbit polyclonal) or Rabbit anti-caspase 3 (1/1000 in milk 5%, Cell signaling 9662S, Rabbit polyclonal) overnight at 4 °C. Then, rinsed membranes are incubated with mouse anti-rabbit horseradish peroxidase (HRP)-conjugated secondary antibody (1/1000 in milk 5%, Santa Cruz sc-2357, Mouse anti-Rabbit IgG-HRP), for 1.5 h at room temperature. Stripped membranes are further incubated with anti-β-actin mouse monoclonal antibody (1/5000 in milk 5%, Sigma A5441, Mouse monoclonal) and donkey anti-mouse horseradish peroxidase (HRP)-conjugated secondary antibody (1/5000 in milk 5%, Jackson 715-035-151). Blots are developed with the chemo-luminescence detection system (ChemiDoc, Bio-Rad^®^), and signal intensity was determined using Quantity One Software (Bio-Rad^®^). The intensity of bands is quantified on ImageLab^®^. The results are expressed as the ratio of protein optic density out of β-Actin optic density and normalized to sham-PBS–PBS, fixed at 100%.

### 4.8. Evaluation of Oligodendrocyte Differentiation by Flow Cytometry in Adolescent Mice

P45 mice are anesthetized and transcardially perfused with 30 mL PBS. Ipsilateral and contralateral brain hemispheres are dissected, and 5 hemispheres are pooled for *n* = 1. After weighting the 5 hemispheres, 3 mL of dissociation enzyme (accutase, A1110501, ThermoFischer Scientific, Waltham, MA, USA) is added to each sample. Each sample is then incubated during 30 min at 37 °C in the GentleMACS^TM^ Octo Dissociator with Heaters (Miltenyi Biotech, Germany). Cell suspension is filtered using 100 µm cell strainers (Dutscher, France). The cell strainers are rinsed with 5 mL of FBS (Fetal Bovine Serum, 6912, CellBiologics, Chicago, IL, USA) diluted at 10% in HBSS (Hank’s Balanced Salt solution, 14170-088, ThermoFischer Scientific, Waltham, MA, USA), and cell suspensions are centrifuged at 500× *g* for 5 min at room temperature. Cell pellet is re-suspended with 5 mL of percoll (P1644, Sigma-Aldrich, St. Louis, MO, USA; diluted 40% in HBSS) and then centrifuged at 650× *g* without break for 25 min at room temperature. Cell pellet is re-suspended in 1mL of staining buffer (1X PBS containing 0.5% BSA). Cells are automatically counted according to the manufacturer’s instructions using the ADAM-MC Cell Counter (NanoEntek Inc., South Korea) associated with the AccuChip kit. After the cell counting, each sample is centrifuged at 650× *g* for 5 min at room temperature. Fc receptors are blocked with anti-mouse CD16/32 for 30 min at 4 °C. Cells are then stained with viability Dye (LIVE/DEAD^TM^—Invitrogen), anti-CD45 antibody for immune cells discrimination and with 1 µg for each antibody: anti-PDGFRα, anti-A2B5, anti-O1 and anti-MBP in flow cytometry staining buffer for 30 min at 4 °C. Details of antibody are summarized in Supplementary Table S2. Cells are washed by adding 2 mL of flow cytometry staining buffer in each sample and centrifuged at 650× *g* for 5 min at room temperature. Samples are run on an BD LSR Fortessa^TM^ (BD Biosciences, Franklin lake, New Jersey, CA, USA) and data are obtained using BD FACSDIVA software (BD Biosciences, Franklin lake, New Jersey, USA). Data are analyzed with FlowJo software (FlowJo, OR, USA). Data beyond mean ±3 standard deviations are excluded. Results are expressed as the percentage of cell per 500 mg of brain tissue. 

### 4.9. Statistical Analysis

Analysis of variance (one-way, two-way ANOVA or three-way ANOVA) followed by Tukey’s post-test is performed for behavioral evaluation, TCC staining, weight intake and immunostaining (GraphPad Prism9 software, CA, USA). Chi2 test is performed for balance beam test. For Western blot, grasping reflex and flow cytometry experiments, the Kruskal–Wallis test is performed followed by Dunn’s post-test. For body temperature monitoring, a two-way ANOVA is performed followed by Tukey’s post-test. For all statistic tests, the significance threshold is set at *p* < 0.05. When sex-depending effects appear, results from males and females are presented through two graphics for easy reading, while males and females performed simultaneously.

## Figures and Tables

**Table 1:** Summary of main results.

Males + Females							
**Experiment**		**HI**			MgSO_4_	MgSO_4_/4-PBA association	
Negative geotaxis, Righting reflex, Cliff aversion, Grasping reflex		Sensorimotor deficits			**++**	**++**	
Immunofluorescence PDGFRα		Oligodendrocyte precursors accumulation in the corpus callosum Oligodendrocyte precursors accumulation in the striatum			**++** **0**	**++** **++**	
TTC		Tissue loss			**++**	**++**	
Social Approach test		Social aproach deficits			**++**	**++**	
Immunofluorescence MBP		Corpus callosum		Corpus callosum surface alteration	**0**	**+**	
		Corpus callosum Thickness (Zone 1)		Corpus callosum thickness alteration	**0**	T	
		Corpus callosum Thickness (Zone 2)			**++**	**++**	
		Corpus callosum Thickness (Zone 3)			**0**	**+**	
		Striatum		Decrease of whiter matter fibers bundles number	**0**	**0**	
		Males			Females		
**Experiment**		**HI**	MgSO_4_	MgSO_4_/4-PBA association	**HI**	MgSO_4_	MgSO_4_/4-PBA association
Mitochondrial apoptotic patwhay		*p* > 0.05			Cleaved cas 3 increase	**0**	T
Balance beam test		locomotion deficits	**+**	**++**	locomotion deficits	**+**	**++**
Foot fault test		*p* > 0.05			Motor deficits	**0**	**++**
NorT		*p* > 0.05			Memory deficits	**0** *	**++**
Flow Cytometry	OPC	*p* > 0.05			Adult OPCs pool alteration	T	**0**
	Pre Myelinating	**0**	**0**	**+**	*p* > 0.05		
	Mature Myelinating	**0**	**0**	T	*p* > 0.05		

Males and females are pooled in the first part of the table since there was no significant sex effect. 0: no effect, +: partial prevention, ++: total prevention, T: tendency to prevent, *: significative improvement of preventive effect; *p* > 0.05: no interaction between factors in ANOVA tests.

## Data Availability

https://drive.univ-rouen.fr/library/c9f15817-2231-4757-a023-6836338bb98f/Ma%20biblioth%C3%A8que/ (accessed on 29 September 2022).

## Supplementary Materials

The following supporting information can be downloaded at: https://www.mdpi.com/article/10.3390/ijms232415947/s1.

## Abbreviations

CP: cerebral palsy; GW: Gestational week; WMI: white matter injury; 4-PBA: 4-phenylbutyrate; MgSO_4_: magnesium sulfate; HDAC: histones deacetylases; HI: hypoxia–ischemia; OL: oligodendrocyte; OPCs: oligodendrocyte progenitor cells; P: postnatal day; Pre-OL: preoligodendrocytes; SB: sodium butyrate.
