# Supplementary material for: Effects of MgSO4 Alone or Associated with 4-PBA on Behavior and White Matter Integrity in a Mouse Model of Cerebral Palsy: A Sex- and Time-Dependent Study
Source: Int J Mol Sci. 2022 Dec 15;23(24):15947. doi: 10.3390/ijms232415947 (PMC9788405; doi:10.3390/ijms232415947)
Supplement: Supplementary file 1 [file ijms-23-15947-s001.zip › ijms-1975430-supplementary.pptx]

## Slide 1
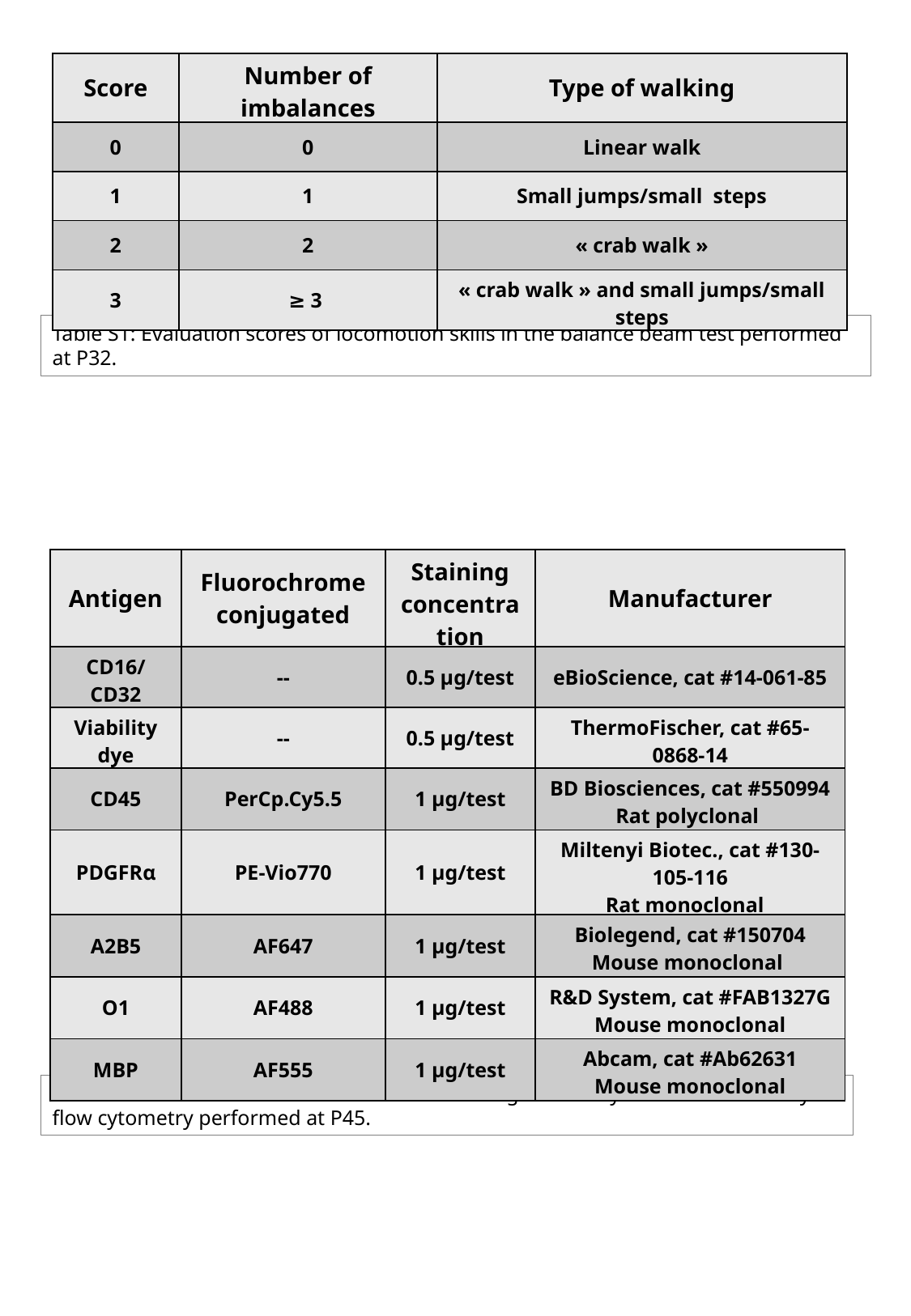

| Score | Number of imbalances | Type of walking |
| --- | --- | --- |
| 0 | 0 | Linear walk |
| 1 | 1 | Small jumps/small steps |
| 2 | 2 | « crab walk » |
| 3 | ≥ 3 | « crab walk » and small jumps/small steps |
Table S1: Evaluation scores of locomotion skills in the balance beam test performed at P32.
| Antigen | Fluorochrome conjugated | Staining concentration | Manufacturer |
| --- | --- | --- | --- |
| CD16/CD32 | -- | 0.5 µg/test | eBioScience, cat #14-061-85 |
| Viability dye | -- | 0.5 µg/test | ThermoFischer, cat #65-0868-14 |
| CD45 | PerCp.Cy5.5 | 1 µg/test | BD Biosciences, cat #550994 Rat polyclonal |
| PDGFRα | PE-Vio770 | 1 µg/test | Miltenyi Biotec., cat #130-105-116 Rat monoclonal |
| A2B5 | AF647 | 1 µg/test | Biolegend, cat #150704 Mouse monoclonal |
| O1 | AF488 | 1 µg/test | R&D System, cat #FAB1327G Mouse monoclonal |
| MBP | AF555 | 1 µg/test | Abcam, cat #Ab62631 Mouse monoclonal |
Table S2: Antibodies utilized for evaluation of oligodendrocyte differenciation by flow cytometry performed at P45.

## Slide 2
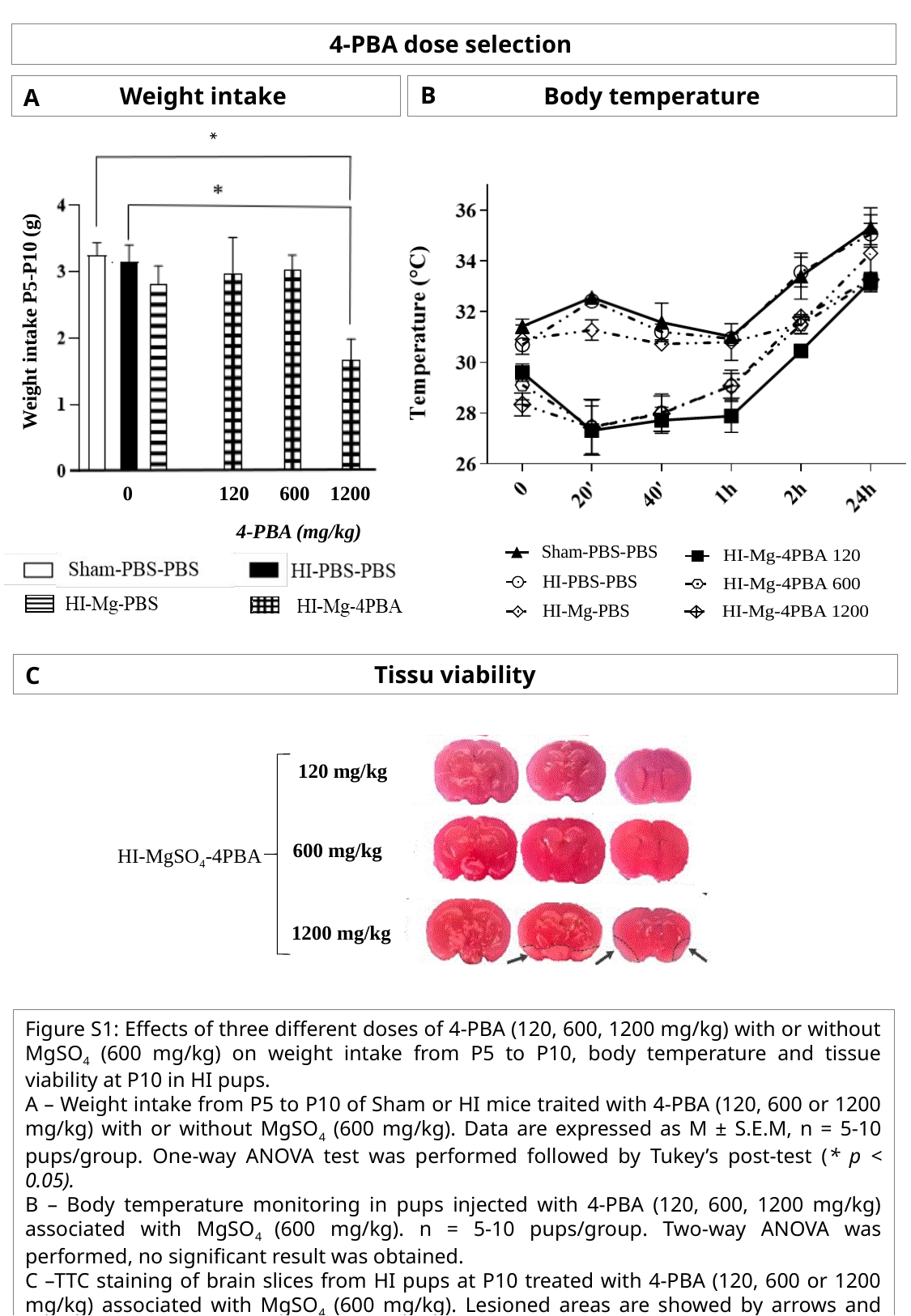

4-PBA dose selection
B
Body temperature
Weight intake
A
*
Weight intake P5-P10 (g)
 0 120 600 1200
4-PBA (mg/kg)
Tissu viability
C
120 mg/kg
600 mg/kg
HI-MgSO4-4PBA
1200 mg/kg
Figure S1: Effects of three different doses of 4-PBA (120, 600, 1200 mg/kg) with or without MgSO4 (600 mg/kg) on weight intake from P5 to P10, body temperature and tissue viability at P10 in HI pups.
A – Weight intake from P5 to P10 of Sham or HI mice traited with 4-PBA (120, 600 or 1200 mg/kg) with or without MgSO4 (600 mg/kg). Data are expressed as M ± S.E.M, n = 5-10 pups/group. One-way ANOVA test was performed followed by Tukey’s post-test (* p < 0.05).
B – Body temperature monitoring in pups injected with 4-PBA (120, 600, 1200 mg/kg) associated with MgSO4 (600 mg/kg). n = 5-10 pups/group. Two-way ANOVA was performed, no significant result was obtained.
C –TTC staining of brain slices from HI pups at P10 treated with 4-PBA (120, 600 or 1200 mg/kg) associated with MgSO4 (600 mg/kg). Lesioned areas are showed by arrows and dotted lines.

## Slide 3
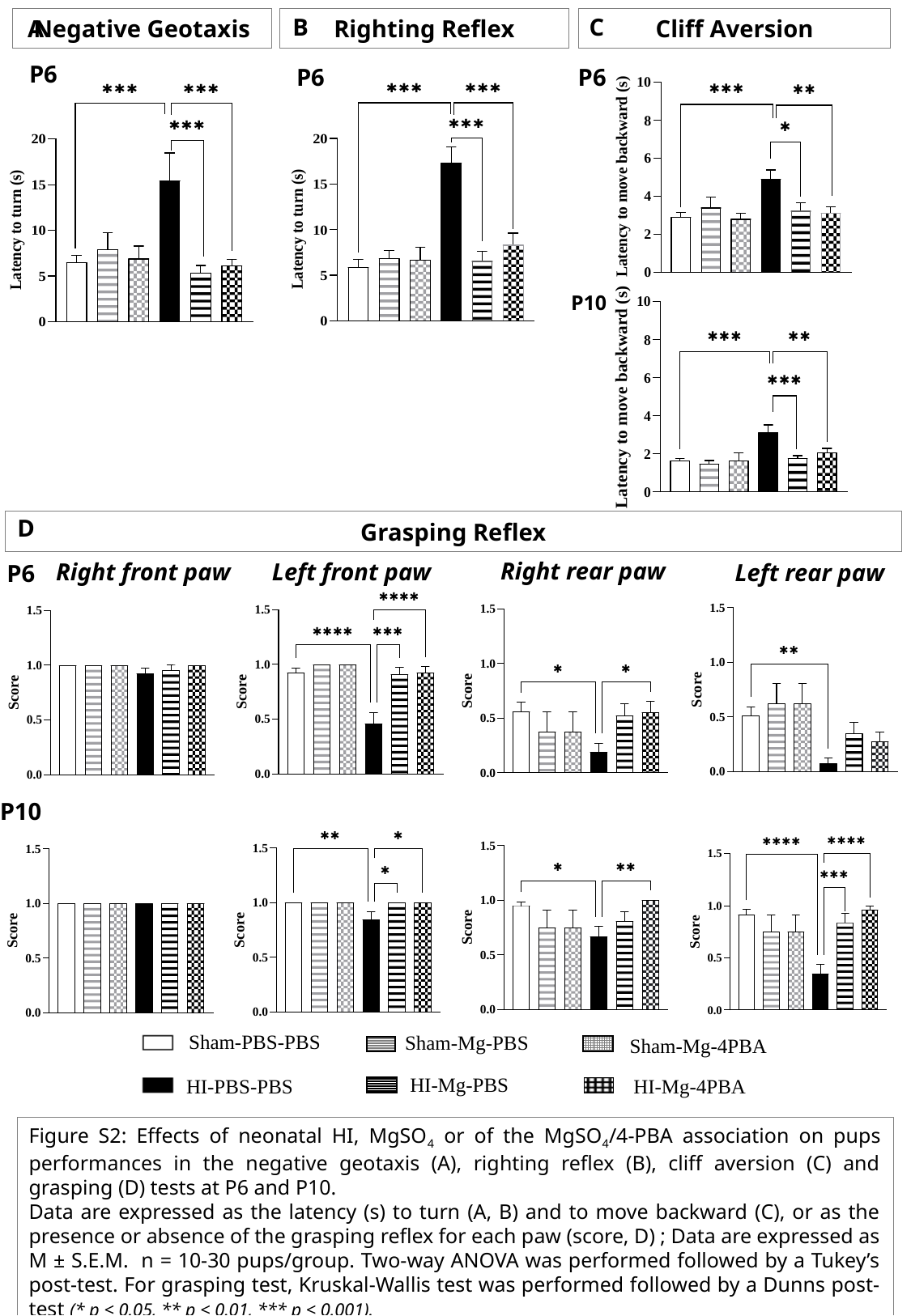

B
C
Negative Geotaxis
Righting Reflex
Cliff Aversion
A
 P6
 P6
 P6
 P10
D
Grasping Reflex
Right rear paw
Right front paw
Left front paw
Left rear paw
P6
P10
Sham-PBS-PBS
Sham-Mg-PBS
Sham-Mg-4PBA
HI-PBS-PBS
HI-Mg-PBS
HI-Mg-4PBA
Figure S2: Effects of neonatal HI, MgSO4 or of the MgSO4/4-PBA association on pups performances in the negative geotaxis (A), righting reflex (B), cliff aversion (C) and grasping (D) tests at P6 and P10.
Data are expressed as the latency (s) to turn (A, B) and to move backward (C), or as the presence or absence of the grasping reflex for each paw (score, D) ; Data are expressed as M ± S.E.M. n = 10-30 pups/group. Two-way ANOVA was performed followed by a Tukey’s post-test. For grasping test, Kruskal-Wallis test was performed followed by a Dunns post-test (* p < 0.05, ** p < 0.01, *** p < 0.001).

## Slide 4
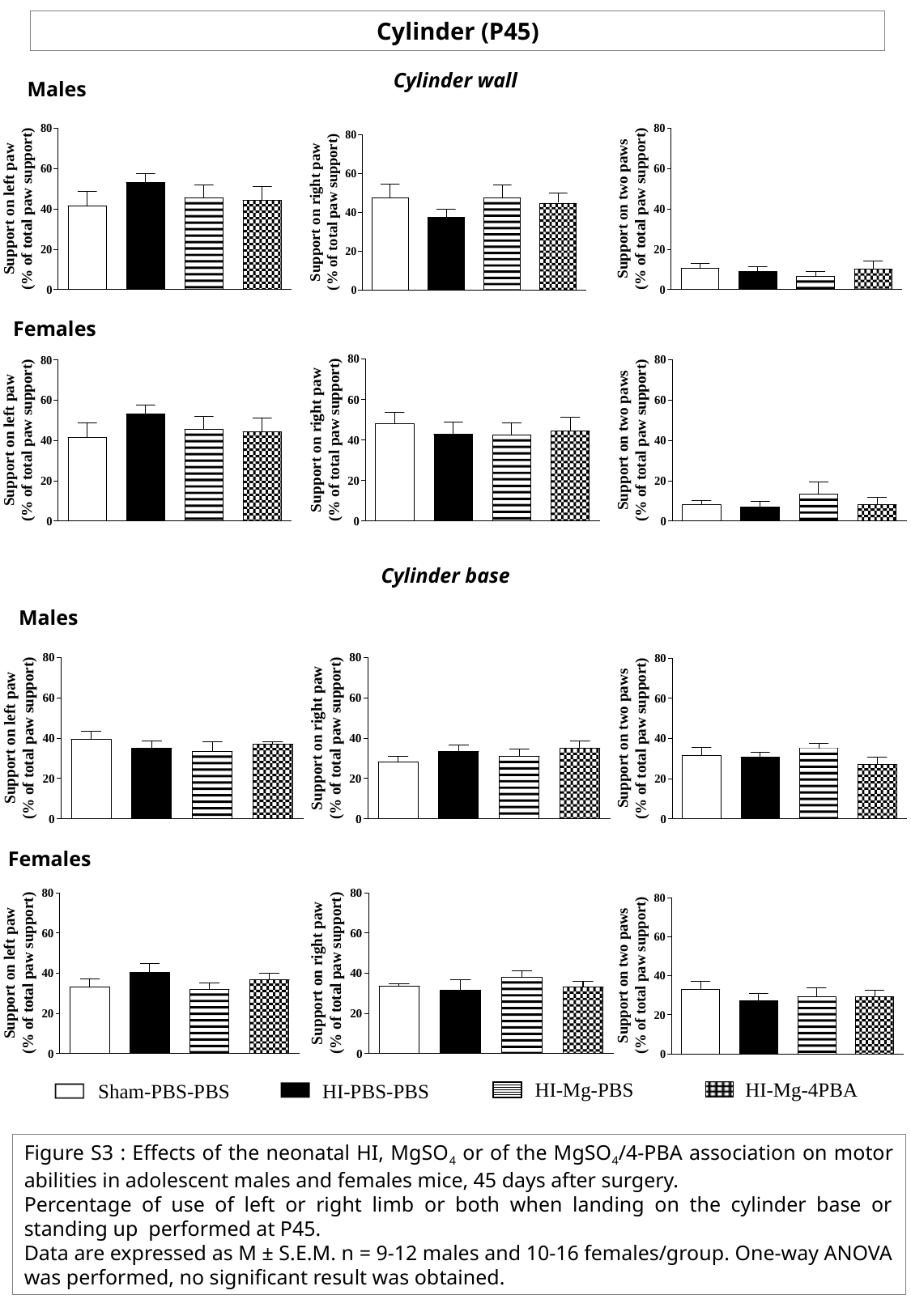

Cylinder (P45)
Cylinder wall
Males
Females
Cylinder base
Males
Females
HI-Mg-4PBA
HI-Mg-PBS
HI-PBS-PBS
Sham-PBS-PBS
Figure S3 : Effects of the neonatal HI, MgSO4 or of the MgSO4/4-PBA association on motor abilities in adolescent males and females mice, 45 days after surgery.
Percentage of use of left or right limb or both when landing on the cylinder base or standing up performed at P45.
Data are expressed as M ± S.E.M. n = 9-12 males and 10-16 females/group. One-way ANOVA was performed, no significant result was obtained.

## Slide 5
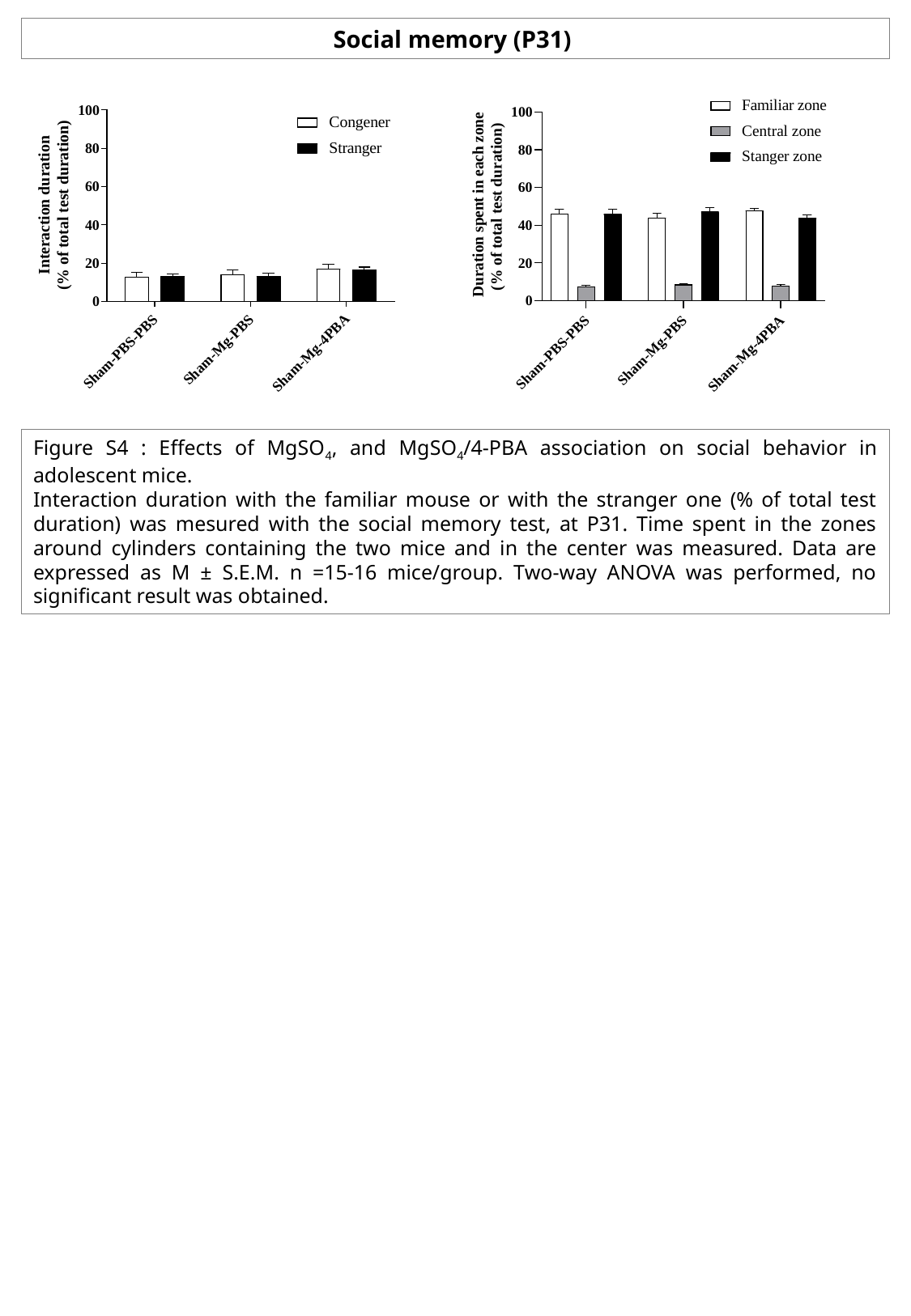

Social memory (P31)
Figure S4 : Effects of MgSO4, and MgSO4/4-PBA association on social behavior in adolescent mice.
Interaction duration with the familiar mouse or with the stranger one (% of total test duration) was mesured with the social memory test, at P31. Time spent in the zones around cylinders containing the two mice and in the center was measured. Data are expressed as M ± S.E.M. n =15-16 mice/group. Two-way ANOVA was performed, no significant result was obtained.

## Slide 6
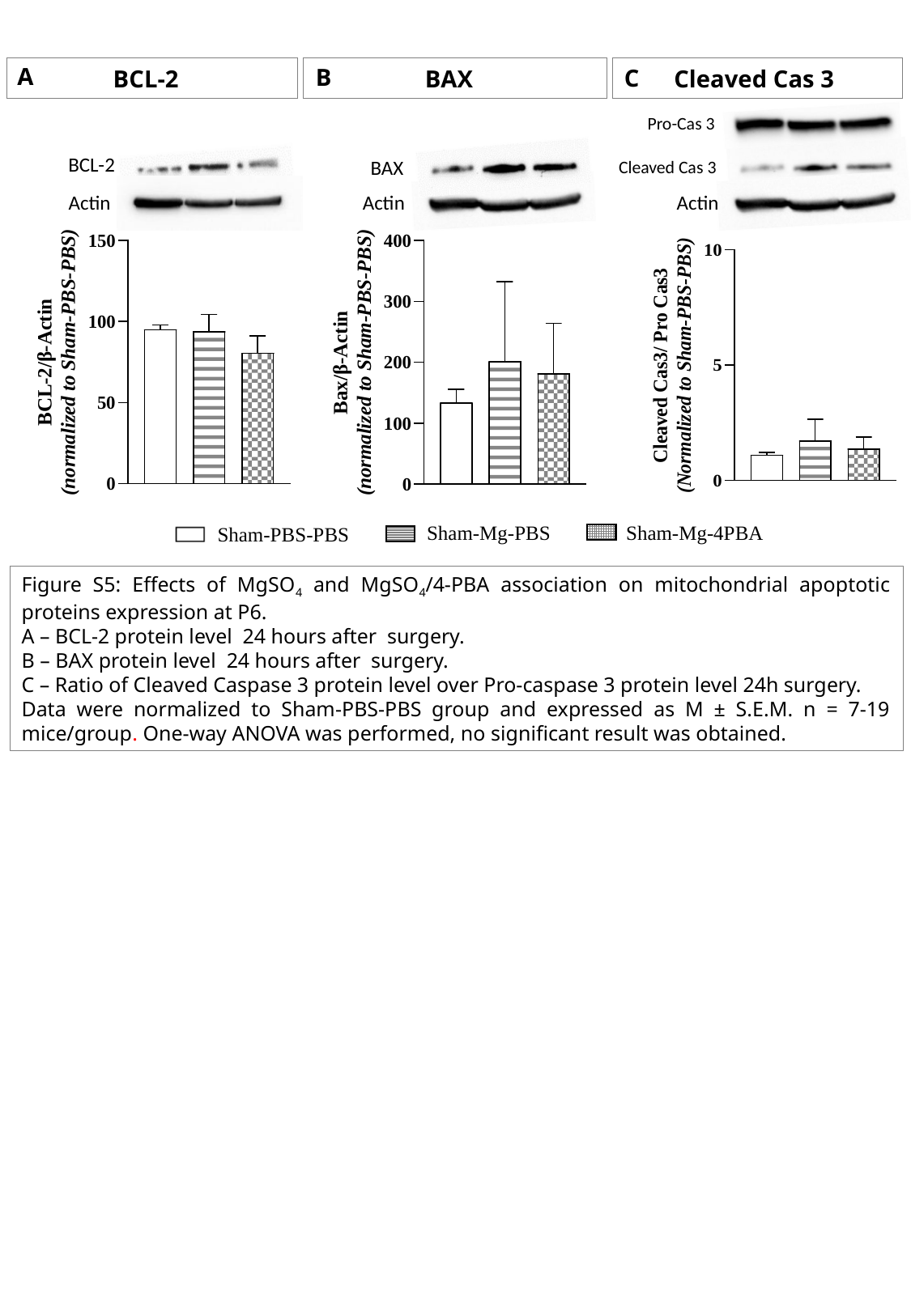

A
B
C
BCL-2
BAX
Cleaved Cas 3
Pro-Cas 3
BCL-2
BAX
Cleaved Cas 3
Actin
Actin
Actin
Sham-Mg-4PBA
Sham-Mg-PBS
Sham-PBS-PBS
Figure S5: Effects of MgSO4 and MgSO4/4-PBA association on mitochondrial apoptotic proteins expression at P6.
A – BCL-2 protein level 24 hours after surgery.
B – BAX protein level 24 hours after surgery.
C – Ratio of Cleaved Caspase 3 protein level over Pro-caspase 3 protein level 24h surgery.
Data were normalized to Sham-PBS-PBS group and expressed as M ± S.E.M. n = 7-19 mice/group. One-way ANOVA was performed, no significant result was obtained.

## Slide 7
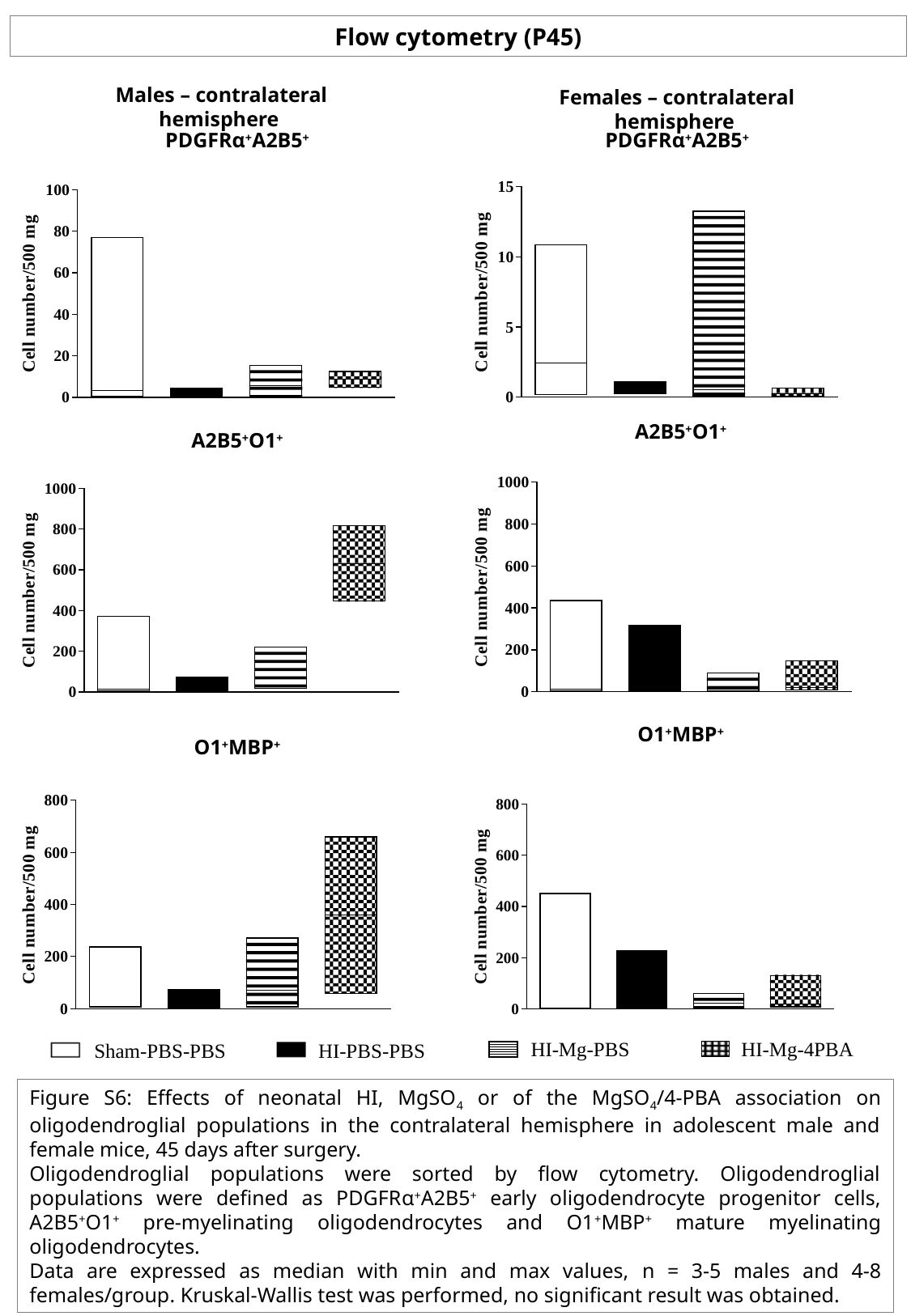

Flow cytometry (P45)
Males – contralateral hemisphere
Females – contralateral hemisphere
PDGFRα+A2B5+
PDGFRα+A2B5+
A2B5+O1+
A2B5+O1+
O1+MBP+
O1+MBP+
HI-Mg-4PBA
HI-Mg-PBS
HI-PBS-PBS
Sham-PBS-PBS
Figure S6: Effects of neonatal HI, MgSO4 or of the MgSO4/4-PBA association on oligodendroglial populations in the contralateral hemisphere in adolescent male and female mice, 45 days after surgery.
Oligodendroglial populations were sorted by flow cytometry. Oligodendroglial populations were defined as PDGFRα+A2B5+ early oligodendrocyte progenitor cells, A2B5+O1+ pre-myelinating oligodendrocytes and O1+MBP+ mature myelinating oligodendrocytes.
Data are expressed as median with min and max values, n = 3-5 males and 4-8 females/group. Kruskal-Wallis test was performed, no significant result was obtained.

## Slide 8
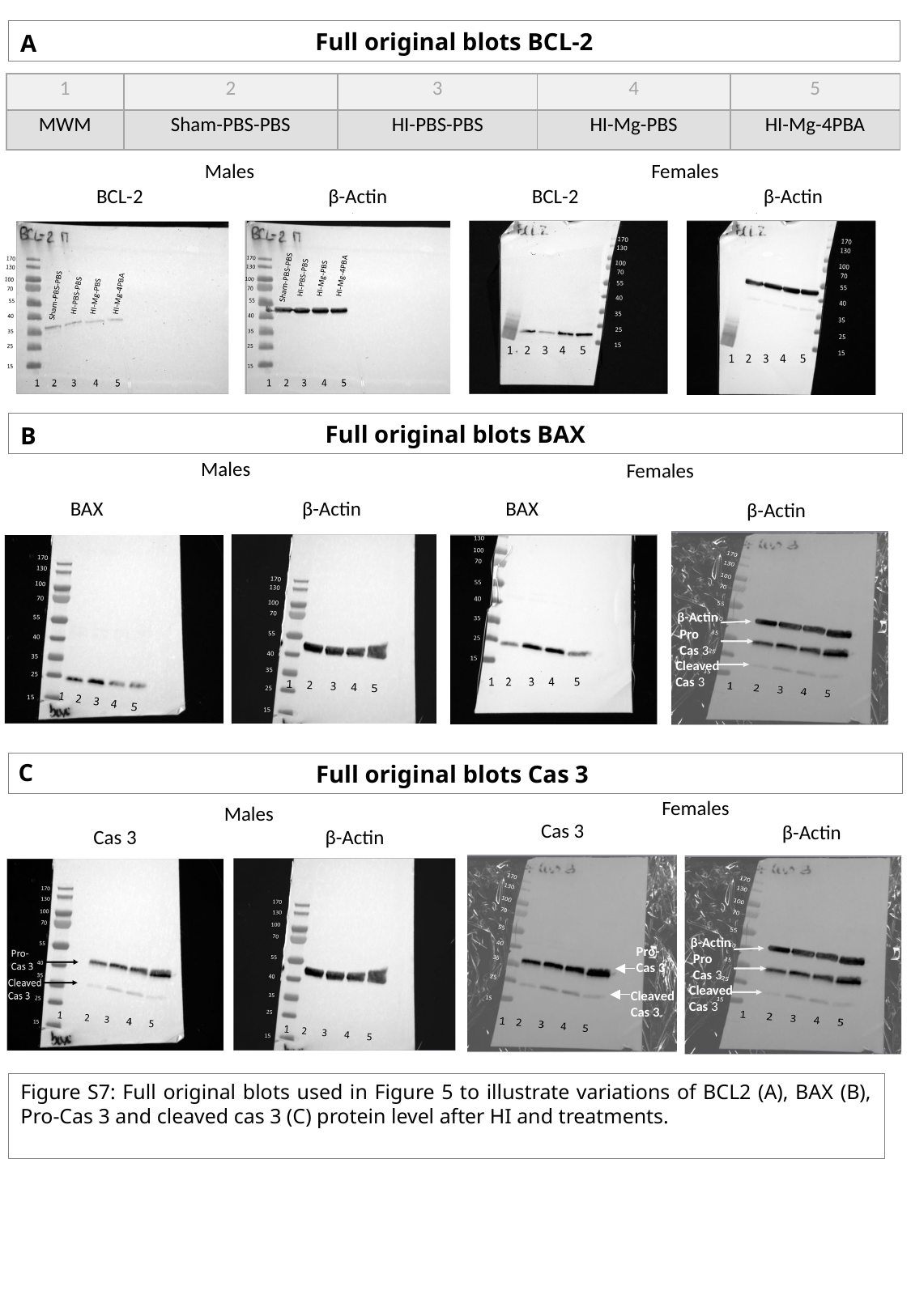

Full original blots BCL-2
A
| 1 | 2 | 3 | 4 | 5 |
| --- | --- | --- | --- | --- |
| MWM | Sham-PBS-PBS | HI-PBS-PBS | HI-Mg-PBS | HI-Mg-4PBA |
Males
Females
BCL-2
β-Actin
BCL-2
β-Actin
Full original blots BAX
B
Males
Females
BAX
β-Actin
BAX
β-Actin
β-Actin
Pro
Cas 3
Cleaved Cas 3
C
Full original blots Cas 3
Females
Males
Cas 3
β-Actin
Cas 3
β-Actin
β-Actin
Pro-Cas 3
Pro
Cas 3
Cleaved Cas 3
CleavedCas 3
Figure S7: Full original blots used in Figure 5 to illustrate variations of BCL2 (A), BAX (B), Pro-Cas 3 and cleaved cas 3 (C) protein level after HI and treatments.

## Slide 9
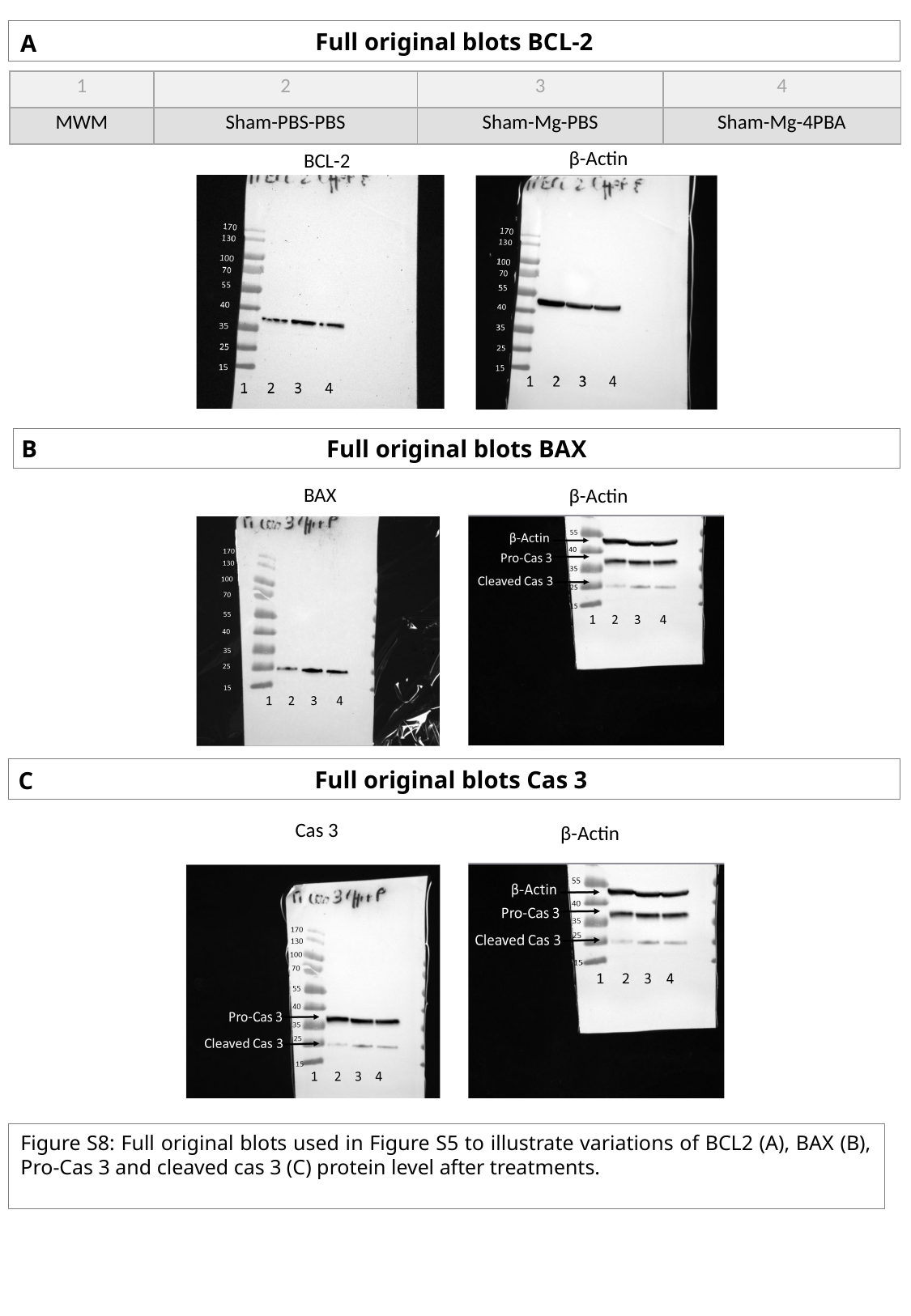

Full original blots BCL-2
A
| 1 | 2 | 3 | 4 |
| --- | --- | --- | --- |
| MWM | Sham-PBS-PBS | Sham-Mg-PBS | Sham-Mg-4PBA |
β-Actin
BCL-2
B
Full original blots BAX
BAX
β-Actin
β-Actin
Pro
Cas 3
Cleaved Cas 3
Full original blots Cas 3
C
Cas 3
β-Actin
β-Actin
Pro-Cas 3
Pro
Cas 3
Cleaved Cas 3
CleavedCas 3
Figure S8: Full original blots used in Figure S5 to illustrate variations of BCL2 (A), BAX (B), Pro-Cas 3 and cleaved cas 3 (C) protein level after treatments.
